# Association of maternal circulating 25(OH)D and calcium with birth weight: A mendelian randomisation analysis

**DOI:** 10.1371/journal.pmed.1002828

**Published:** 2019-06-18

**Authors:** William D. Thompson, Jessica Tyrrell, Maria-Carolina Borges, Robin N. Beaumont, Bridget A. Knight, Andrew R. Wood, Susan M. Ring, Andrew T. Hattersley, Rachel M. Freathy, Debbie A. Lawlor

**Affiliations:** 1 Institute of Biomedical and Clinical Science, College of Medicine and Health, University of Exeter, Exeter, United Kingdom; 2 MRC Integrative Epidemiology Unit at the University of Bristol, Bristol, United Kingdom; 3 Population Health Science, Bristol Medical School, University of Bristol, Bristol, United Kingdom; 4 Avon Longitudinal Study of Parents and Children, NIHR Bristol Biomedical Research Centre, University of Bristol, Bristol, United Kingdom; 5 Bristol NIHR Biomedical Research Centre, Bristol, United Kingdom; University of Manchester, UNITED KINGDOM

## Abstract

**Background:**

Systematic reviews of randomised controlled trials (RCTs) have suggested that maternal vitamin D (25[OH]D) and calcium supplementation increase birth weight. However, limitations of many trials were highlighted in the reviews. Our aim was to combine genetic and RCT data to estimate causal effects of these two maternal traits on offspring birth weight.

**Methods and findings:**

We performed two-sample mendelian randomisation (MR) using genetic instrumental variables associated with 25(OH)D and calcium that had been identified in genome-wide association studies (GWAS; sample 1; *N* = 122,123 for 25[OH]D and *N* = 61,275 for calcium). Associations between these maternal genetic variants and offspring birth weight were calculated in the UK Biobank (UKB) (sample 2; *N* = 190,406). We used data on mother–child pairs from two United Kingdom birth cohorts (combined *N* = 5,223) in sensitivity analyses to check whether results were influenced by fetal genotype, which is correlated with the maternal genotype (r ≈ 0.5). Further sensitivity analyses to test the reliability of the results included MR-Egger, weighted-median estimator, ‘leave-one-out’, and multivariable MR analyses. We triangulated MR results with those from RCTs, in which we used randomisation to supplementation with vitamin D (24 RCTs, combined *N* = 5,276) and calcium (6 RCTs, combined *N* = 543) as an instrumental variable to determine the effects of 25(OH)D and calcium on birth weight. In the main MR analysis, there was no strong evidence of an effect of maternal 25(OH)D on birth weight (difference in mean birth weight −0.03 g [95% CI −2.48 to 2.42 g, *p* = 0.981] per 10% higher maternal 25[OH]D). The effect estimate was consistent across our MR sensitivity analyses. Instrumental variable analyses applied to RCTs suggested a weak positive causal effect (5.94 g [95% CI 2.15–9.73, *p* = 0.002] per 10% higher maternal 25[OH]D), but this result may be exaggerated because of risk of bias in the included RCTs. The main MR analysis for maternal calcium also suggested no strong evidence of an effect on birth weight (−20 g [95% CI −44 to 5 g, *p* = 0.116] per 1 SD higher maternal calcium level). Some sensitivity analyses suggested that the genetic instrument for calcium was associated with birth weight via exposures that are independent of calcium levels (horizontal pleiotropy). Application of instrumental variable analyses to RCTs suggested that calcium has a substantial effect on birth weight (178 g [95% CI 121–236 g, *p* = 1.43 × 10^−9^] per 1 SD higher maternal calcium level) that was not consistent with any of the MR results. However, the RCT instrumental variable estimate may have been exaggerated because of risk of bias in the included RCTs. Other study limitations include the low response rate of UK Biobank, which may bias MR estimates, and the lack of suitable data to test whether the effects of genetic instruments on maternal calcium levels during pregnancy were the same as those outside of pregnancy.

**Conclusions:**

Our results suggest that maternal circulating 25(OH)D does not influence birth weight in otherwise healthy newborns. However, the effect of maternal circulating calcium on birth weight is unclear and requires further exploration with more research including RCT and/or MR analyses with more valid instruments.

## Abstract 2

William Thompson and colleagues, using mendelian randomisation methods, reveal no association between levels of maternal circulating vitamin D and birth weight of newborns.

## Author summary

**Why was this study done?:**

- Birth weight that is lower or higher than average has been associated with poor health outcomes across the life span, including infant mortality, cardiovascular disease, and type 2 diabetes.
- If we can identify modifiable maternal factors in pregnancy that are causally related to birth weight, we may be able to reduce the number of babies that are born with lower or higher than optimal birth weight. This may in turn help to reduce the associated poor health outcomes.
- Previous studies have suggested that higher maternal 25(OH)D and calcium in pregnancy are associated with higher birth weight. However, many of those studies used conventional multivariable regression in observational studies and may be subject to residual confounding. Few of them estimated the size of the effect of either maternal 25(OH)D or calcium levels on birth weight.

**What did the researchers do and find?:**

- We estimated the effects of maternal gestational 25(OH)D and calcium levels on offspring birth weight using mendelian randomisation, a method that uses genetic data to overcome certain limitations of traditional observational studies—for example, residual confounding.
- We analysed genetic data on 190,406 women from the UK Biobank who reported the birth weight of their first child, along with the results from published studies of genetic associations with 25(OH)D and calcium levels in 122,123 and 61,275 individuals, respectively.
- We checked that the results were not biased by offspring genotype using data from two UK birth cohorts, the Avon Longitudinal Study of Parents and Children (ALSPAC; *n* = 4,576 mother–child pairs) and Exeter Family Study of Childhood Health (EFSOCH; *n* = 647 mother–child pairs), respectively.
- To strengthen our causal understanding, we triangulated the mendelian randomisation results with findings from randomised controlled trials in which we used randomised status (to vitamin D or calcium supplementation) as an instrumental variable to estimate the effect of 25(OH)D or calcium on birth weight.
- We found no evidence of a strong effect of maternal 25(OH)D on birth weight.
- We found inconsistent evidence of effects of maternal calcium on birth weight.

**What do these findings mean?:**

- Our findings do not support using vitamin D supplementation during pregnancy or preconceptually to influence offspring birth weight.
- The effect of calcium on birth weight is still unclear and needs further investigation in well-powered genetic studies and/or well-conducted randomised controlled trials.

## Introduction

Infants with lower or higher birth weight (BW) than average are at an increased risk of neonatal mortality and morbidity [1]. BW is also inversely associated with some adverse adult health outcomes, including coronary heart disease [2], type 2 diabetes [3], poor cognitive ability [4], and several types of cancer [5], with most of these associations being linear across most of the BW distribution. BW is an indicator of conditions in utero and may be influenced by modifiable factors in the maternal circulation. For example, there is evidence that higher maternal fasting glucose is causally related to greater fetal growth and higher BW [6,7], which increases the risk of complications during delivery. However, relatively little is known about the causal influences of other maternal factors. More evidence is required on how modifying the in utero environment might influence BW and associated health outcomes.

Maternal gestational circulating 25(OH)D [8] and calcium [9] may be modifiable risk factors that impact fetal growth and hence BW. Several observational studies using conventional multivariable regression analyses suggest positive associations of maternal 25(OH)D and calcium with infant BW [10–13]; however, these results might be explained by residual confounding. Systematic reviews of randomised controlled trials (RCTs) of gestational supplementation with vitamin D [14] or calcium [15] have suggested that this supplementation increases BW. However, the authors of the latest vitamin D supplementation systematic review concluded that most of the trials were small and of low quality, and the difference in mean BW was small and unlikely to be of clinical or public health importance [16]. For the calcium supplementation systematic review, the authors noted that for most of the trials, there was a low risk of bias based on a score that did not include intention to treat as one of the risk-of-bias criteria. However, there were high levels of heterogeneity in the results between the trials, bringing into question the clinical importance of calcium supplementation on BW [15].

Mendelian randomisation (MR) is a method in which genetic variants associated with a modifiable exposure are used as instrumental variables to estimate the causal effect of the exposure on an outcome [17]. As genetic variants are fixed at conception and are generally not associated with classical confounders, MR is less susceptible to bias resulting from reverse causation and residual confounding [17]. We have previously performed an MR study on the effects of maternal adiposity related exposures on BW, finding a positive effect of body mass index (BMI) and blood glucose and an inverse causal effect of systolic blood pressure on BW [6]. In that study, there was evidence of a possible positive causal effect of 25(OH)D on BW; however, the CIs were wide and included the null value [6]. A possible causal association of maternal circulating calcium with infant BW was not explored in that study, and to the best of our knowledge, there have been no MR studies of that association. The aim of this study was to use MR to explore whether there are causal effects of maternal circulating 25(OH)D and calcium on BW and, if so, what the magnitude those effects are. With the release of new UK Biobank (UKB) data [18], we have a substantially increased sample size in comparison to the earlier MR study of 25(OH)D, as well as access to more genetic instruments for 25(OH)D [19], both of which will increase statistical power and hence effect estimate precision. We complement our MR analyses by triangulating results with findings from instrumental variable analyses applied to RCTs of supplementation with vitamin D or calcium [20].

## Methods

The analyses plan was developed by RMF, DAL, WDT, and MCB, prior to any analyses beginning. It was acted on by WDT and MCB. The analysis plan has not been published but was informally recorded in meeting notes. We made one change to the overall study plans after completing analyses; we undertook risk-of-bias assessment of the RCTs with a focus on factors that might mean instrumental variable assumptions were violated. This was motivated by differences in results comparing MR to instrumental variables in RCTs, particularly in relation to the effects of calcium on BW. We made one change to the plan following reviewers’ comments; we undertook two multivariable MR analyses to adjust for potential confounders. These were (1) multivariable MR of the association of 25(OH)D with BW adjusting (genetically) for maternal height and (2) partial multivariable MR of the association of calcium with BW adjusting (genetically) for maternal educational level.

**Fig 1:**
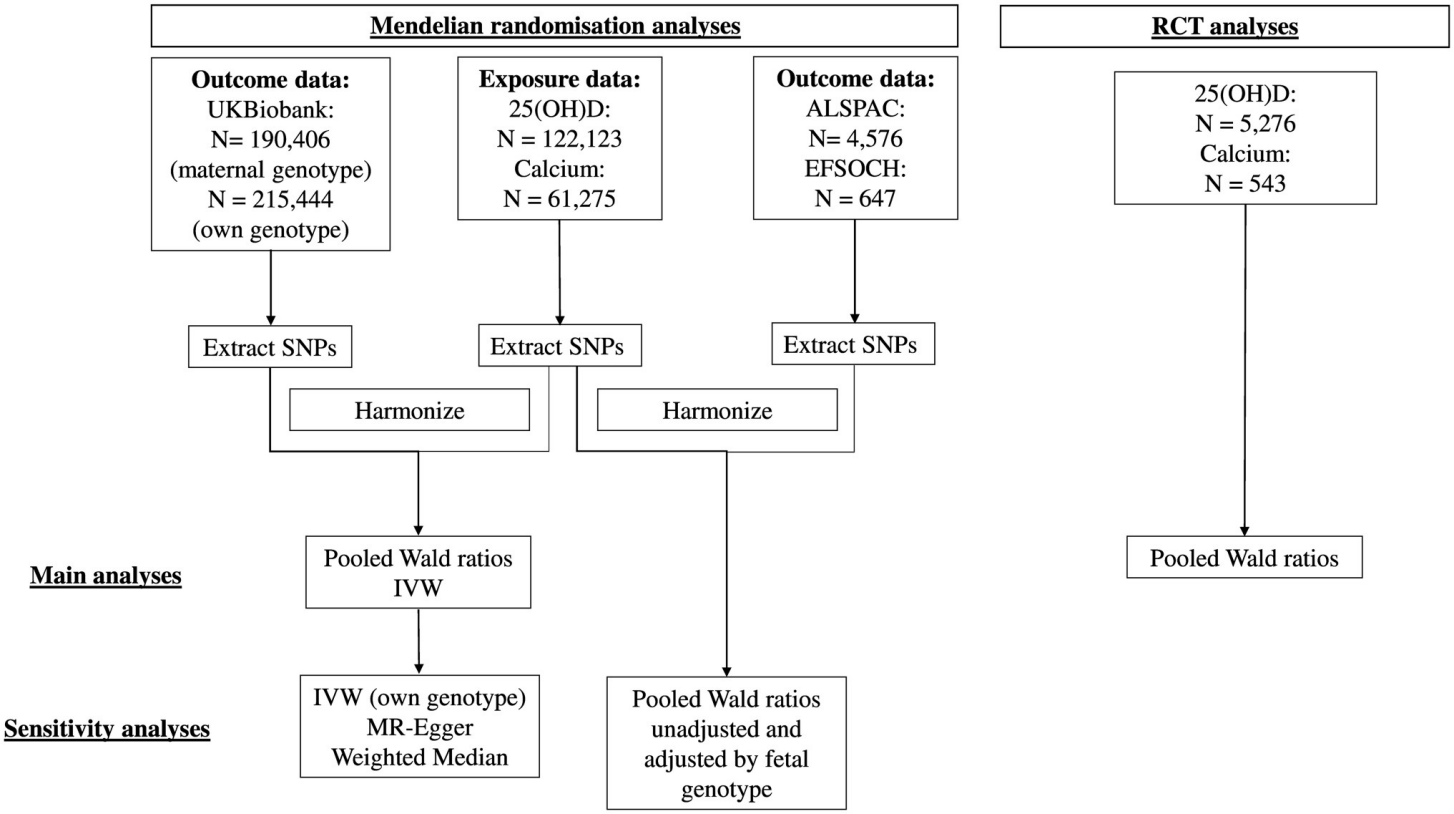
Summary of methods and data contributing to this study. ALSPAC, Avon Longitudinal Study of Parents and Children; EFSOCH, Exeter Family Study of Childhood Health; IVW, inverse variance–weighted; MR, mendelian randomisation; RCT, randomised controlled trial; SNP, single-nucleotide polymorphism.

The study design and different data sources are summarised in Fig 1, with Table 1, S1, S2, S3 and S4 Text, S1, S2, S3 and S4 Tables, S1, S2 and S3 Figs providing more data on each study that has contributed to this paper. Details of participant consent are described in S1 Text. Ethical approval for data extraction from all of the cohorts used in this study was granted from the appropriate authorities (for more details, see S1 Text). This study is reported according to the Strengthening the Reporting of Observational Studies in Epidemiology (STROBE) guideline (S1 Checklist).

**Table 1:** Characteristics of the studies used to obtain 25(OH)D/calcium SNPs’ (genetic instrumental variables) effects on offspring birth weight.

	Study	Average/Typical Value of Study
Country	UK Biobank	United Kingdom
	ALSPAC	United Kingdom
	EFSOCH	United Kingdom
Offspring years of birth	UK Biobank	1954–2011
	ALSPAC	1991–1993
	EFSOCH	2000–2004
Number of participants	UK Biobank	190,406
	ALSPAC	4,576
	EFSOCH	647
Maternal age, years	UK Biobank	25.9 (5.0)
	ALSPAC	29.0 (4.6)
	EFSOCH	30.4 (5.2)
Maternal BMI, kg/m^2^	UK Biobank	27.07 (5.03)
	ALSPAC	22.91 (3.72)
	EFSOCH	24.03 (4.32)
Maternal height, cm	UK Biobank	162.5 (6.1)
	ALSPAC	164.5 (6.7)
	EFSOCH	165.0 (6.3)
Birth weight, g	UK Biobank	3,227 (476)
	ALSPAC	3,495 (471)
	EFSOCH	3,514 (475)
Gestational age, weeks	UK Biobank	NA
	ALSPAC	39.8 (1.3)
	EFSOCH	40.1 (1.2)
Offspring sex, % male	UK Biobank	NA
	ALSPAC	49
	EFSOCH	52
Maternal systolic blood pressure, mmHg	UK Biobank	141 (24)
	ALSPAC	133 (13)
	EFSOCH	NA
Mothers smoking, %^a^	UK Biobank	12
	ALSPAC	15
	EFSOCH	14
Townsend deprivation index^b^	UK Biobank	−1.66 (2.86)
	ALSPAC	NA
	EFSOCH	0.23 (3.29)
Educational attainment: mothers with a university degree, %	UK Biobank	47
	ALSPAC	14
	EFSOCH	NA
Western diet^c^ (SD)	UK Biobank	−0.087 (0.98)
	ALSPAC	NA
	EFSOCH	NA
Age of assessment for age of first birth, years	UK Biobank	58.0 (7.8)
	ALSPAC	NA
	EFSOCH	NA

^a^In ALSPAC and EFSOCH, this is the percentage of women who smoked during pregnancy; for UK Biobank, it is the percentage of women who smoke.

^b^An area deprivation index that takes summary data on deprivation measures from the census for a defined small geographical area (percentage of households without a motor vehicle, percentage of households with more than one person per room, percentage of households not owner-occupied, and percentage of residents who are unemployed), converts them to SD scores across all areas of the UK, and then sums them to give a relative area deprivation value, such that a higher score indicates greater deprivation for the area compared to the UK as a whole [21]; in UK Biobank, these reflect adult area of residence deprivation based on the postcode provided by the participants at their baseline assessment (aged 40–60 years).

^c^Western diet is a principal component of variation in reported diet in UK Biobank. Variation in diet was measured using a dietary questionnaire [22].

Abbreviations: ALSPAC, Avon Longitudinal Study of Parents and Children; BMI, body mass index; EFSOCH, Exeter Family Study of Childhood Health.

### Mendelian randomisation

We used two-sample summary data MR to explore the effect of maternal circulating vitamin D (25[OH]D) and calcium levels on offspring BW [23]. We used (1) summary data from published genome-wide association (GWA) studies (GWAS) for the associations of genetic variants (single-nucleotide polymorphisms [SNPs]) with 25(OH)D [19,24] or calcium [25] (sample 1) and (2) summary data for the associations of SNPs with BW from UKB [18] (sample 2). Summary data from two UK birth cohorts, the Avon Longitudinal Study of Parents and Children (ALSPAC) [26] and Exeter Family Study of Childhood Health (EFSOCH) [27], were generated for use in sensitivity analyses to explore bias due to fetal genotype. In all studies, we excluded participants of non–white European origin (S2 Text describes how ethnicity was defined in each study). Following these exclusions, we included 190,406 women from UKB who had valid data on BW of first child and GWAS data, 4,576 mother–offspring pairs from ALSPAC, and 647 mother–offspring pairs from EFSOCH who all had offspring BW and maternal and offspring GWAS data.

### BW and serum 25(OH)D measurement

The UKB is a study of 502,655 participants [28]. Female participants (*N* = 273,495) were also asked to report the BW of their first child. Female participants that reported having a multiple first birth were excluded from our analyses (*N* = 1,364). A total of 216,839 women with a singleton pregnancy for their first child reported the BW of their first child. Values were reported to the nearest whole pounds and were converted to kilograms by multiplying by 0.454 for our analyses. When women reported the BW of the first child at multiple time points (*N* = 11,353), we used the mean of all measures after excluding any women with a difference of >1 kg between any two measures (*N* = 31). We further excluded from the whole sample any women who reported the BW of their first child < 2.2 kg or > 4.6 kg (*N* = 6,333). This was done to reduce bias from reporting errors and, in relation to those < 2.2 kg, to exclude extreme preterm births, given that we do not have information on gestational age. BW of first child was regressed against the women’s reported age at first birth and UKB assessment centre location to reduce heterogeneity in reporting bias by these characteristics. Residuals from that regression model were then standardised to a mean of 0 and an SD of 1, with the standardised residuals being used in all analyses and final results converted back to grams. The analyses were done on standardised residuals to reduce the amount of computing power needed when doing the initial GWA analyses, and so the summary GWAS data that we used for our main analyses were already in this standardised format. UKB participants’ (women and men) reports of their own BWs were used in sensitivity analyses described below, and similar methods of exclusion and use of standardised (on age at assessment and centre) residuals, with conversion of results back to grams, were used for own BW (see S3 Text for further details).

In ALSPAC and EFSOCH, BW was extracted (in grams) from obstetric clinical records at the time of birth, which occurred between 1991 and 1992 (mean [SD] age of mothers 29 [4.6] years) in ALSPAC [26] and between 2000 and 2004 (mean age of mothers 30.4 [5.2] years) in EFSOCH [27].

The GWAS of 25(OH)D and calcium that we have used in our MR analyses were undertaken on adult European-origin (nonpregnant) women and men. Our MR analyses assume that the magnitude of gene instrument variable-25(OH)D (or calcium) association from those studies are the same in women during pregnancy. We were able to test this for 25(OH)D in ALSPAC, in which 25(OH)D was measured during pregnancy in mothers, using methods that have previously been reported [29] (see S4 Text). Neither 25(OH)D nor calcium were measured during pregnancy in UKB or EFSOCH; calcium was not measured in ALSPAC.

### Genotyping

For UKB, we analysed data from the May 2017 release of imputed genetic data, which have been extensively described elsewhere [30]. Given the reported technical error with non-HRC imputed variants [31], we focused exclusively on the set of approximately 40 million imputed variants from the HRC reference panel.

To account for population structure and relatedness, a linear mixed model implemented in BOLT-LMM v2.3 [32] was used to perform genome-wide association (GWA) analysis of BW in the UKB sample. Only autosomal SNPs that were common (MAF > 1%), in Hardy Weinberg equilibrium (HWE; *p*-value > 1 × 10^−6^), passed QC in all 106 batches, and were present on both genotyping arrays were included in the genetic relationship matrix (GRM). For the GWA analyses of BW of the first child (i.e., using the maternal genotype), the genotyping array and genotyping release (interim versus full) were included as covariates in the regression model. For the GWAS of participants’ own BW (see below under ‘Exploring violation of MR assumptions’ for the rationale behind these analyses), genotyping array, age at baseline, and sex were adjusted for in all models.

In both ALSPAC and EFSOCH, the SNPs used in this study (see below) were taken from genome-wide imputed data that had been completed for both the mothers and their offspring (fetal genotype). In ALSPAC, maternal data were obtained from the Illumina 610 Quad Array, and fetal data were obtained from the Illumina 550 Quad Array. In EFSOCH, maternal and fetal data were obtained from the Illumina Infinium HumanCoreExome-24. For both ALSPAC and EFSOCH, genotype data were imputed against Haplotype Reference Consortium HRC v1.1 reference panel after quality control (MAF > 1%, HWE > 1 × 10^−6^, sex mismatch, kinship errors, and 4.56 SD from the cluster mean of any subpopulations cluster) [26,33].

### SNP selection and summary data for SNP–25(OH)D and SNP–calcium associations

We searched for the largest well-conducted GWAS to identify genetic variants (SNPs) that could be used as instrumental variables for circulating 25(OH)D and calcium and to obtain summary data of genetic instrumental variable (SNP) associations with 25(OH)D and calcium for use in our two-sample MR.

For 25(OH)D, we used summary association results for SNPs identified in two GWAS [19,24], with the largest of these including 79,366 participants from 31 studies in discovery analyses and 42,757 participants from two studies in replication analyses. In our main analyses, we used seven SNPs, which were not in linkage disequilibrium, from either of the GWAS that had a *p*-value of 5 × 10^−8^ in discovery analyses and were replicated. Two of the SNPs discovered in the largest GWAS (rs3755967 and rs17216707) were different from, but in the same loci as, two SNPs identified in an earlier, more commonly used GWAS (rs2282679 and rs6013897, respectively) [34] that have been commonly used as instruments of 25(OH)D in previous MR analyses. We measured linkage disequilibrium between these SNPs in white Europeans (CEU) using LDLink [35,36] and found that both were >0.5, suggesting the SNPs in each pair are tagging the same variant. In additional analyses, we separately conducted two analyses with genetic instruments hypothesised to be involved in 25(OH)D synthesis (three SNPs) and 25(OH)D metabolism (two SNPs) [37].

For calcium, we used summary association results for SNPs identified in a GWAS of 39,400 participants [25] from 19 studies in discovery analyses and 21,875 participants from 11 studies as replication. We used seven SNPs, which were not in linkage disequilibrium, that were associated with calcium levels at a *p*-value of 5 × 10^−8^ in discovery analysis and were replicated.

Further details of the 25(OH)D and calcium GWAS are provided in S1 Table, and a list of the SNPs used in our MR analyses, together with their allele frequencies and per-allele associations, for 25(OH)D and calcium are provided in S2 Table.

For UKB, the summary results of associations between SNPs and first-child BW (maternal genotype) or own BW (own genotype) were extracted from the GWAS results (see above for details on how each GWAS was conducted). For ALSPAC and EFSOCH, individual-level SNP data were extracted and summary data were generated using multivariate linear regression of the SNPs against BW (adjusting for gestational age and the child’s sex).

To make sure that the outcome data (BW) and exposure data (25[OH]D and calcium) were comparable, the SNPs’ effects were harmonised to the 25(OH)D/calcium-raising alleles using procedures that have previously been described [38].

### Statistical analysis

The main and sensitivity two-sample MR methods are summarised in Table 2. In all analyses, we estimated the effect of a 10% increase in 25(OH)D on BW in grams and the effect of 1 SD increase in calcium on BW in grams, these units reflecting the units of 25(OH)D and calcium used in the published GWAS. The value of a 10% increase in 25(OH)D will vary depending on the ‘starting point’. In the gestational measures of 25(OH)D in ALSPAC, the median level of 25(OH)D is 61.8 nmol/l, the 25th percentile is 46.1 nmol/l, and the 75th percentile is 81.6 nmol/l; this makes a 10% increase from these points equivalent to 6.2 nmol/l, 4.6 nmol/l, and 8.2 nmol/l, respectively. The accepted range of calcium in a healthy population is between 8.5 mg/dl and 10.5 mg/dl [39], and dividing that range by four, we estimated that the SD of calcium is 0.5 mg/dl (approximately 0.3 mmol/l).

**Table 2:** Summary of the four methods used for MR analysis.

Name of Method	Wald Ratio (Meta-analysis) [40]	Inverse Variance–Weighted [41]	MR-Egger [41]	Weighted Median [42]
Assumption	There is no unbalanced horizontal pleiotropy.	There is no unbalanced horizontal pleiotropy.	That the effect of the genetic instrument is not correlated with any pleiotropic effect of the instrument on the outcome.	Less than 50% of the weight in the analyses comes from invalid instruments.
Equation	$Wald\;Ratio=\frac{\beta_{y|z}}{\beta_{x|z}}$	$\beta_{IVW=}\frac{\sum_{j=1}^{J}E_{j}^{2}\sigma e_{j}^{-2}\beta_{j}}{\sum_{j=1}^{J}E_{j}^{2}\sigma e_{j}^{-2}}$	$\beta_{MR}=\frac{\rho_{j-\beta_{0}}}{E_{j}}$	$\rho_{j}=100(S_{j}-\frac{w_{j}}{2})$
Notes on equation	β_y|z_ is the SNP’s effect on the outcome, and β_x|z_ is the SNP’s effect on the exposure. Wald ratios for each SNP were pooled using fixed-effect meta-analysis with inverse-variance weights.	β_j_ is the ratio method estimate for each genetic instrument, σej−2 is the standard error of the genetic variants effect on outcome, and Ej2 is the genetic instruments effect on the exposure.	β_0_ is the intercept, E_j_ is the genetic instruments effect on the exposure, and ρ_j_ is the genetic instruments effect on the outcome.	Multiple ratio estimates, or β_j_, are calculated, and the median percentile value is chosen. Each percentile value is weighted. S_j_ is the sum of the weights up to the given genetic instrument, ρ_j_ is the percentile value, and w_j_ is the weight given to the genetic instrument.

Abbreviations: MR, mendelian randomisation; SNP, single-nucleotide polymorphism.

The main effects were calculated in all three studies (UKB, ALSPAC, and EFSOCH) using two methods: fixed-effect meta-analysis of Wald ratios [40] of the seven SNPs for 25(OH)D or the seven SNPs for calcium and the inverse variance–weighted (IVW) instrumental variable method [41] (Table 2).

Wald ratios were calculated by dividing each SNP’s effect on BW by the same SNP’s effect on the exposure (25[OH]D or calcium). Standard errors were calculated by dividing the standard error of the SNP’s effect on BW by each SNP’s effect on the exposure. The ratios for each SNP were then pooled using fixed-effect meta-analysis. I^2^ and leave-one-out analysis were used to explore between SNP heterogeneity in their MR results (which, if present, may be due to one or more of the SNPs being an invalid instrumental variable) [43].

For the IVW analysis, linear regression of the weighted (by inverse of their variance) SNPs’ associations with BW against the SNPs’ association with maternal circulating 25(OH)D or calcium was performed [41]. In IVW regression analyses, the intercept is forced through zero, making the regression coefficient comparable to the pooled Wald ratio effect estimate. In the presence of heterogeneity, standard errors are larger for IVW compared to pooled Wald ratios. For our analysis, we estimated standard errors using a fixed-effects model and CIs using a t-distribution.

### Exploring possible violations of MR assumptions

Both the Wald ratio method and IVW instrumental variable analysis assume that (1) the SNPs being used are robustly associated with maternal circulating 25(OH)D and calcium, (2) the SNPs are not related to confounders of the associations of 25(OH)D and/or calcium with BW, and (3) the SNPs have no effect on BW other than through 25(OH)D and calcium (also known as the exclusion restriction criterion). In MR studies, horizontal pleiotropy is a common cause of violation of this assumption. This would occur if our genetic instrument for 25(OH)D or calcium influenced other factors, separately to 25(OH)D or calcium, and these other factors influence BW independently of 25(OH)D or calcium. If this were the case, then the estimate of effect that we assumed was due to, e.g., 25(OH)D would be the sum of a 25(OH)D effect and the effect on the outcome of any other (pleiotropic) effects. The Wald ratio and IVW approaches complement each other, with determining Wald ratios for each SNP providing an opportunity to explore between SNP heterogeneity and IVW being closely related and comparable to one of our sensitivity analyses (MR-Egger) used to test possible horizontal pleiotropy (Table 2).

One possible source of bias is via the fetal genotype [44]. Maternal genetic variants that influence 25(OH)D and calcium will be associated with the distributions of the same genetic variants in the fetus (as mothers may transmit these alleles to their offspring), and if any of these genetic variants affect fetal growth independently of an effect of maternal circulating 25(OH)D/calcium (for example, if fetal 25[OH]D or calcium influence fetal growth), there will be an association between maternal SNPs and offspring BW that is not via the mother’s gestational 25(OH)D or calcium. We tested this possible source of bias in two ways. First, we adjusted the maternal SNP with offspring BW association for fetal genotype in a total of 5,223 genotyped mother–child pairs from the ALSPAC and EFSOCH studies. The Wald ratio results were estimated separately for each of these two cohorts and then pooled using a fixed-effect meta-analysis. Second, we used IVW to estimate the effect of own 25(OH)D or calcium on own BW (with a total of 215,444 adult women and men reporting their own BW) in UKB for comparison with the effect estimates of maternal 25(OH)D or calcium on offspring BW. Stronger effects of own (fetal) 25(OH)D/calcium on their BW (compared with maternal gestational circulating levels of these on offspring BW) would suggest the possibility of our main MR analyses of maternal 25(OH)D/calcium levels on offspring BW being biased by fetal effects (assuming that measurement error in offspring BW and own BW are similar).

We performed three additional tests to investigate possible violations of MR assumptions: MR-Egger [41] and weighted-median estimator [42], which were only used in UKB (which we considered our main analysis cohort and which has adequate statistical power for these analyses), and exploring SNP associations with confounders in UKB, ALSPAC, and EFSOCH (further details of these approaches are provided in S5 Text).

### Instrumental variable analysis applied to RCTs

Instrumental variable methods can be applied to RCTs to quantify the causal effect estimate of the intermediate that the randomised treatment is assumed to influence [45]. For example, here we used RCTs of randomisation to vitamin D supplements to quantify the effect of circulating 25(OH)D on BW. This differs from the original aim and analyses of these RCTs, which was to determine the causal effect of the supplements. These analyses are similar to MR, except here the instrumental variable is randomised status. This approach has the same underlying assumptions as all instrumental variable analyses, including MR. However, we assume that the key sources of violation of these assumptions will differ between the RCT and MR analyses (e.g., in the RCTs, concealment of randomisation and intention-to-treat analyses will be important, whereas in MR horizontal pleiotropy due to linkage disequilibrium will be important). Under this assumption, if results from our MR and RCT analyses are consistent with each other, this increases the likelihood that this is the correct causal effect [20].

We used data from recent systematic reviews and meta-analyses of supplements versus placebo in pregnant women for both 25(OH)D [16] and calcium [15] to identify individual RCTs that could be used in our instrumental variable analyses applied to RCTs. As different RCTs used different doses, type of supplement (e.g., vitamin D2 or D3), or mode of delivery (e.g., oral or injection), and because to date relatively little work has used instrumental variables in RCTs to test causal effects, we a priori decided we would use a one-sample instrumental variable approach and only include RCTs that provided both the difference in mean BW and difference in mean 25(OH)D/calcium by randomised arm. Each individual RCT in both reviews was searched to identify those that provided difference in mean BW and 25(OH)D/calcium. This resulted in us being able to include 24 (56%) out of 43 RCTs from the most recent pregnancy vitamin D supplementation systematic review (published 2017) and 6 (26%) out of 23 RCTs included in the most recent calcium supplementation systematic review (2015); all other RCTs had either no information on differences in mean BW or differences in mean 25(OH)D/calcium.

Two of the authors (WDT and DAL) independently extracted the weighted mean differences in 25(OH)D and BW by trial randomised arm (25[OH]D supplement or placebo/other control), together with their respective CI values, from 24 RCTs that presented results for both of these (*N* = 5,276 mother–offspring pairs) [16] (S3 Table). Similarly, for calcium, two authors (WDT and M-CB) independently extracted mean differences and CIs for 6 RCTs (543 mother–offspring pairs) [15] (S4 Table). Standard errors were calculated by each of the independent abstractors, and a third author (RMF) checked consistency between the abstractors with any discrepancy resolved by discussion between four authors, WDT, DAL, M-CB, and RMF. One of the calcium RCTs [46] provided a range of the mean difference, which we treated as the 95% CIs when calculating the standard error.

We calculated the Wald ratio estimate for each RCT by dividing the difference in mean BW by the difference in mean 25(OH)D (or calcium) and then pooled these using a fixed-effect meta-analysis and tested for between-study heterogeneity using the I^2^ statistic and leave-one-out analysis. For the vitamin D supplementation, RCTs’ differences in mean 25(OH)D were in nmol/l, and for the calcium supplementation, RCTs’ differences in mean calcium were in mg/dl. In order to make the instrumental variable analyses in RCT results comparable with our two-sample MR results, for 25(OH)D we assumed that a 10% difference was equivalent to 6.2 nmol/l (the value for a 10% difference around the median of the distribution of pregnancy 25[OH]D in ALSPAC), and we multiplied the Wald ratio estimates by 6.2 to scale the results so that they represented the difference in mean BW per 10% increase in 25(OH)D. We did the same with calcium but used 0.5 (0.5 mg/dl being the 1 SD difference value used in our MR analysis) to scale results so that they represented the difference in mean BW per 1 SD of calcium.

## Results

The characteristics of included participants from UKB, ALSPAC, and EFSOCH are shown in Table 1. The SNP-outcome associations for UKB, ALSPAC, and EFSOCH are shown in S5, S6 and S7 Tables.

### MR and instrumental variable analysis in RCTs do not support a clinically important effect of 25(OH)D on BW

**Fig 2:**
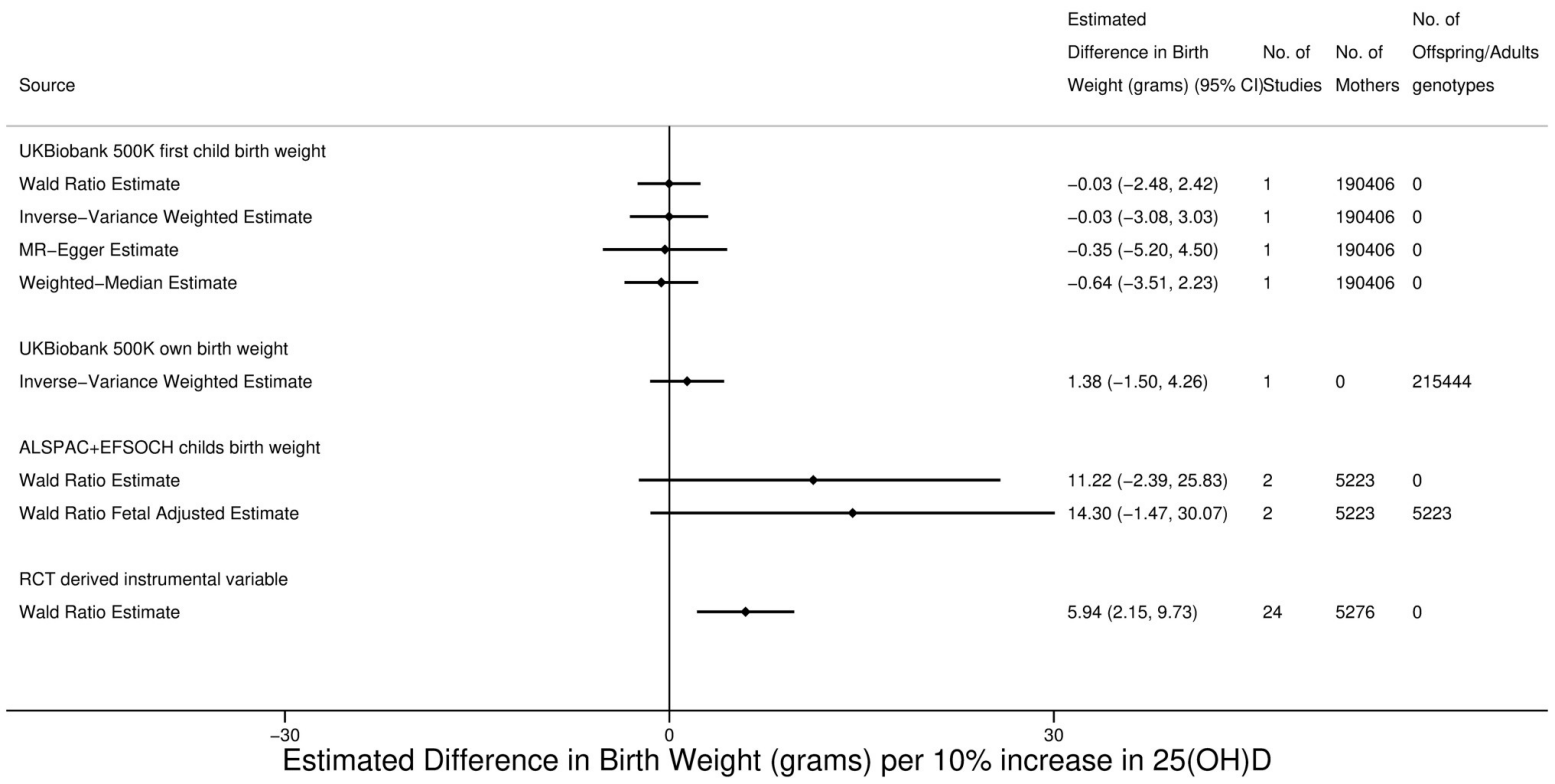
Causative effect estimates for maternal 25(OH)D on birth weight. ALSPAC, Avon Longitudinal Study of Parents and Children; EFSOCH, Exeter Family Study of Childhood Health; MR, mendelian randomisation; RCT, randomised controlled trial.

Our two-sample MR results provide no strong evidence of an effect of maternal circulating 25(OH)D on offspring BW, with consistent null findings in the main Wald ratio and IVW analyses and also with the MR-Egger and weighted-median results using the UKB data (Fig 2). There was no strong evidence for marked heterogeneity between the Wald ratio estimates for each SNP (I^2^ = 0.0%), and results were consistent in leave-one-out analysis with the main results (i.e., no SNPs removed) and with each other (S4 Fig). MR effects in ALSPAC and EFSOCH were weakly positive but with wide CIs that included the null value (Fig 2). The effect estimates were the same with and without adjustment for fetal genotype in ALSPAC and EFSOCH, and there was no strong evidence that own 25(OH)D influenced own BW in UKB (Fig 2). Our main MR effect estimate in UKB was −0.03 g (95% CI −2.48 to 2.42 g, *p* = 0.981) per 10% increase in maternal circulating 25(OH)D. However, the RCT instrumental variable effect was 5.94 g (95% CI 2.15–9.73, *p* = 0.002) per 10% increase in maternal circulating 25(OH)D (Fig 2). There was no strong evidence for marked heterogeneity between the instrumental variable in RCT estimates (I^2^ = 16.2%), and results were consistent with the main overall result and with each other in leave-one-out analysis (S5 Fig).

The difference between the MR and instrumental variable analyses in RCTs is small, and the CIs for the two results overlap, suggesting that they are statistically consistent. To explore this small difference further, we undertook post hoc risk-of-bias assessment of the 24 RCTs. S8 Table summarises the results of this risk-of-bias assessment. Most studies were small, with the numbers randomised being between 16 and 1,134 and only three of the 24 RCTs including more than 200 participants. When it was reported (16 of the 24 trials), loss to follow-up ranged from 1% to 31%. Only two of the RCTs (12% of participants) had definitely undertaken intention-to-treat analyses, and 12 (54% of studies) had used random sequence and concealed allocation to treatment groups.

Though there were only a small number of genetic instruments, and thus limited ability to test potential biases from horizontal pleiotropy and weak instruments, there was no strong evidence that 25(OH)D synthesis or metabolism had an effect on BW (S6 Fig).

### MR and instrumental variable analyses applied to RCTs give conflicting results on the effect of calcium on BW

**Fig 3:**
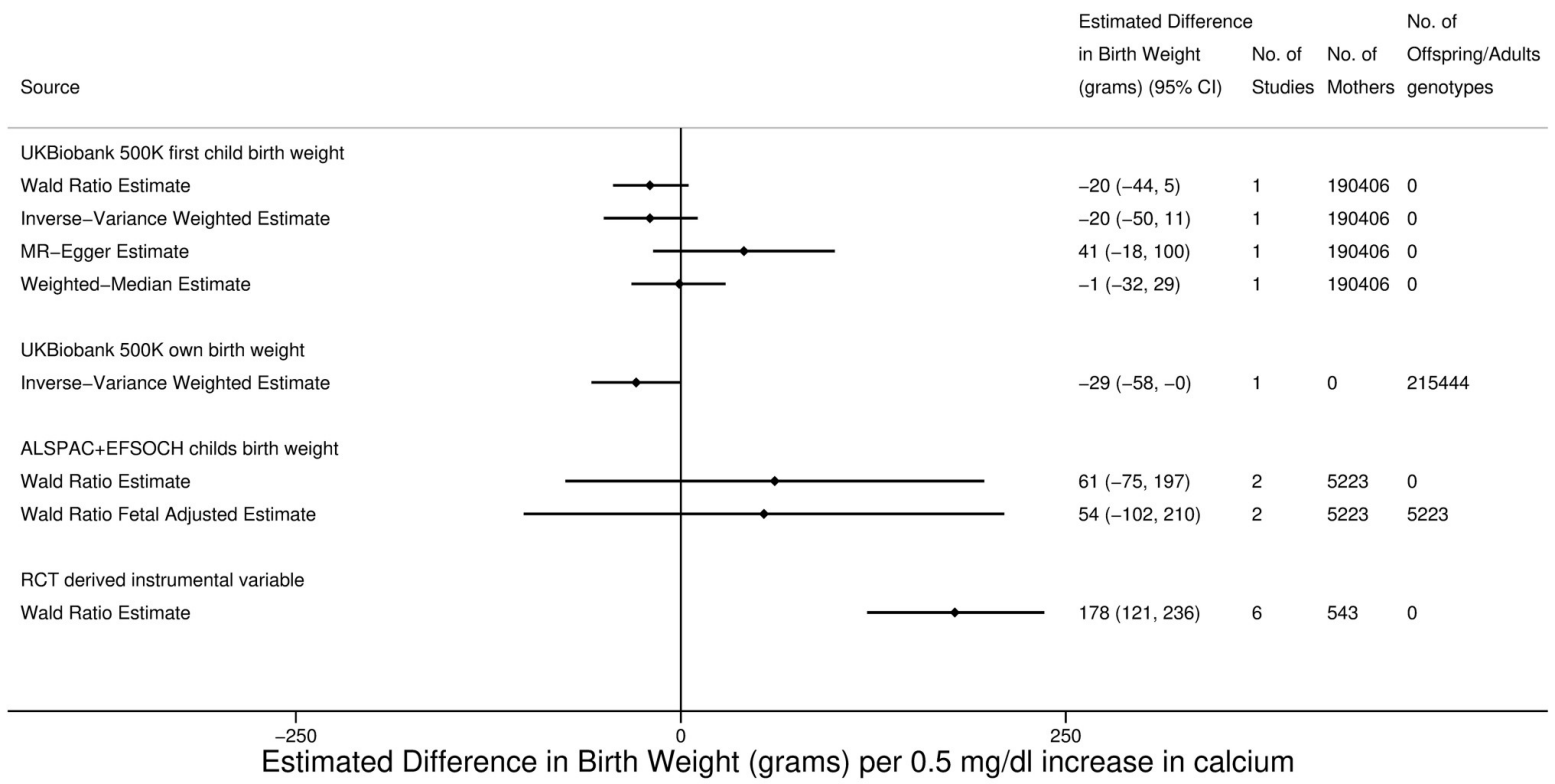
Causative effect estimates for maternal calcium on birth weight. ALSPAC, Avon Longitudinal Study of Parents and Children; EFSOCH, Exeter Family Study of Childhood Health; MR, mendelian randomisation; RCT, randomised controlled trial.

Our main two-sample MR results provide no strong evidence of an effect of maternal circulating calcium on offspring BW, with consistent null findings across the Wald ratio, IVW, and weighted-median results using UKB. However, there was evidence of horizontal pleiotropy, with the MR-Egger intercept being −2 g (95% CI −4 to −0.5 g, *p* = 0.025) and the effect estimate suggesting a possible modest effect of 41 g (95% CI −18 to 100 g, *p* = 0.132) per 1 SD increase in maternal circulating calcium, compared to the weak inverse effect in the IVW and Wald ratio estimates of −20 g (95% CI −44 to 5 g, *p* = 0.116) (Fig 3). There was also evidence for heterogeneity between the Wald ratio estimates for each SNP (I^2^ = 62.4%), and removing one of the SNPs (rs1801725) strengthened the inverse effect estimate to −61 g (95% CI −99 to −23 g, *p* = 0.002) per 1 SD increase in maternal circulating calcium, with removal of the other six SNPs resulting in estimates close to the main result and consistent with each other (S7 Fig). The Wald ratio for only the rs1801725 SNP was 10 g (95% CI −22 to 41 g, *p* = 0.558) per 1 SD increase in maternal circulating calcium. There was no evidence that fetal genotype influenced the results, as results were similar with or without adjustment for it, and the IVW analyses for adults’ own BW gave a similar result to the IVW analyses for first child’s BW (Fig 3). The RCT instrumental variable effect on calcium suggested a strong positive effect that was inconsistent with any of the MR estimates (including MR-Egger): 178 g (95% CI 121–236 g, *p* = 1.43 × 10^−9^) change in BW per 1 SD increase in maternal circulating calcium (Fig 3). There was no strong evidence for heterogeneity between the RCT instrumental variable results (I^2^ = 0.0%), but the main result and leave-one-out analyses had wide CIs (S8 Fig). Given the marked difference in the instrumental variable results in RCTs compared with any of the MR analyses, we (post hoc) looked for potential bias within the specific RCTs that we were able to include in our one-sample instrumental variable analyses of calcium on BW. Of the six included RCTs, three had fewer than 100 participants (*N*s = 23 to 72), and the two largest RCTs (*N* = 120 and 274), together with two other studies, did not use intention-to-treat analysis (S9 Table), which could result in biased effect estimates. The second-largest study (*N* = 120) was also judged by the authors of the systematic review to have high risk of bias (or it was noted that there was insufficient information to determine risk) across all of the specific domains they assessed (S9 Table).

### Validity of the 25(OH)D and calcium genetic instruments

The magnitude of the 25(OH)D SNPs association with maternal circulating 25(OH)D in ALSPAC was similar to the magnitudes reported in the source GWAS for five of the seven SNPs. There were differences for rs8018720 (which was weakly inversely rather than positively associated with 25[OH]D in ALSPAC) and for rs117913124 (a weaker positive difference in ALSPAC when compared to source GWAS) (Table 3). Despite these differences, we found no evidence of heterogeneity between the Wald ratios, and leave-one-out analyses found no evidence of an outlier effect (see above).

**Table 3:** The 25(OH)D instruments’ effect on exposure in GWAS source and ALSPAC.

SNP	Nearby Genes	Effect Allele	Effect Allele Frequency (GWAS Reported)	GWAS	No. of Participants in GWAS	Difference in Mean 25(OH)D per Allele From GWAS, in natural log (log) nmol/l (95% CI)	No. of Participants in ALSPAC	Difference in Mean 25(OH)D per Allele From ALSPAC, in log nmol/l (95% CI)
rs3755967	*GC*	C	0.72	Jiang and colleagues [19]	79,366	0.089 (0.084–0.094)	4,874	0.069 (0.048–0.091)
rs117913124	*CYP2R1*	G	0.975	Manousaki and colleagues [24]	42,274	0.21 (0.19–0.23)*	4,874	0.079 (0.018–0.139)
rs10741657	*CYP2R1*	A	0.4	Jiang and colleagues [19]	79,366	0.031 (0.027–0.035)	4,874	0.01 (−0.010 to 0.029)
rs12785878	*DHCR7*	T	0.75	Jiang and colleagues [19]	79,366	0.036 (0.032–0.04)	4,874	0.049 (0.026–0.071)
rs10745742	*AMDHD1*	T	0.41	Jiang and colleagues [19]	79,366	0.017 (0.013–0.021)	4,874	0.029 (0.009–0.049)
rs8018720	*SEC23A*	G	0.27	Jiang and colleagues [19]	79,366	0.017 (0.012–0.022)	4,874	−0.037 (−0.062 to −0.011)
rs17216707	*CYP24A1*	T	0.79	Jiang and colleagues [19]	79,366	0.026 (0.021–0.031)	4,874	0.020 (−0.005 to 0.046)

*This result differs from that reported by Manousaki and colleagues. Their result was in units of SDs of 25(OH)D in log nmol/L. We converted that value to natural logged nmol/l by estimating the value of an SD of log transformed nmol/L of 25(OH)D in the ALSPAC study.

Abbreviations: ALSPAC, Avon Longitudinal Study of Parents and Children; *AMDHD1*, Amidohydrolase Domain Containing 1; *DHCR7*, 7-Dehydrocholesterol reductase; GWAS, genome-wide association studies; SNP, single-nucleotide polymorphism.

Neither the 25(OH)D nor the calcium genetic instrumental variable–weighted allele scores were associated with observed confounders in the UKB, ALSPAC, or EFSOCH, with the exceptions of maternal height, education, and Townsend deprivation index (TDI). The score for 25(OH)D was negatively associated with height in the UKB, positively associated with height, and negatively associated with TDI in EFSOCH and showed no association in ALSPAC; the score for calcium was negatively associated with educational level and positively associated with TDI in UKB (S10 Table). Given these findings, and on advice from one of the reviewers, we undertook further analyses, using multivariable MR [47], to explore whether maternal height might have masked a positive effect of 25(OH)D on BW (maternal height could be a masking confounder in these results, as it is inversely associated with 25[OH]D but positively relates to BW) and whether maternal education confounded the MR effect estimate of maternal calcium on BW (further details available in S5 Text). Results from the multivariable IVW MR analyses were consistent with those from the unadjusted IVW MR analyses, for both the full adjustment of height for 25(OH)D–BW results and the partial maternal education calcium–BW results (S11 Table).

## Discussion

This study triangulated two approaches (two-sample MR and instrumental variables applied to RCTs) assessing the effects of maternal circulating 25(OH)D and calcium on BW. Across the main and all sensitivity MR analyses, we found no evidence that maternal 25(OH)D has an important effect on BW, but applying instrumental variable analyses to RCTs, there was evidence of a weak positive effect. Findings for maternal circulating calcium were inconsistent across methods and sensitivity analyses, making it difficult for us to conclude from the data used here what the effect of maternal circulating calcium on BW is.

Although the instrumental variable applied to RCT analyses for 25(OH)Ds effect suggested a weak positive effect of 5.94 g (95% CI 2.15–9.73, *p* = 0.002) higher BW per 10% increase in 25(OH)D, this might be exaggerated by limitations of the original RCTs (S8 Table and recent systematic review [16]) and is so small that it is unlikely to be of clinical or public health importance. Our MR analyses are largely in European-origin populations, whereas the RCTs were predominantly in South Asian or Middle Eastern populations, and several were also in those with low 25(OH)D levels at the start of pregnancy. These population differences may also have contributed to differences between the two approaches, though it is notable that the RCTs suggest little evidence of an effect on BW even in these populations at high risk of vitamin D insufficiency. One of the proposed mechanisms underlying the hypothesis that maternal circulating 25(OH)D results in higher BW is by increasing offspring bone mineral density (BMD). However, a recent large, well-conducted RCT found no evidence of an effect of maternal vitamin D_3_ supplementation on offspring neonatal BMD (assessed within 2 weeks of birth) [48]. When combined with our own findings, there is therefore little evidence to suggest that maternal circulating 25(OH)D influences BW via increases in BMD. A recent MR study suggested no evidence of circulating 25(OH)D affecting the risk of preeclampsia [49], which is consistent with results of a meta-analysis of RCTs of vitamin D supplementation on preeclampsia [16]. That same meta-analysis found no strong evidence that randomisation to vitamin D supplementation influenced gestational diabetes risk, when restricting analyses to RCTs with least risk of bias [16]. As preeclampsia and gestational diabetes affect BW [6], these findings are consistent with our results suggesting maternal 25(OH)D does not affect BW.

It is possible that low circulating 25(OH)D levels are not a suitable indicator of vitamin D deficiency, as 1,25(OH)2D is the biologically active form of vitamin D and remains within reference limits even when circulating 25(OH)D levels are low, suggesting valid genetic and RCT instruments associated with 1,25(OH)2D levels could give different results. However, we are not aware of such instruments, and circulating 25(OH)D has a longer half-life in the body, making it a more stable measurement and possibly a better indicator of long-term vitamin D exposure [50]. Furthermore, previous observational studies that underpin the hypothesis that vitamin D levels are importantly related to BW, and a large number of other health outcomes, have used 25(OH)D as the marker of exposure.

Although the main MR analyses did not support a causal effect of maternal calcium on offspring BW, sensitivity (MR-Egger and leave-one-out) analyses were not completely consistent with the main MR effect estimates, and the instrumental variable analyses applied to RCTs were markedly different from any of the MR results. There are a number of possible reasons why we might have found these differences. The MR estimate may be biased by masking horizontal pleiotropy, which both our MR-Egger and leave-one-out analyses suggest. Thus, the MR-Egger effect estimate suggests a modest positive effect with wide CIs, and the leave-one-out analyses suggest that the heterogeneity between individual SNP Wald ratios is driven by one SNP, rs1801725, which when removed results in an inverse association (again suggesting masking pleiotropy). rs1801725 is in the calcium-sensing receptor (*CASR*) gene, which codes for the calcium-sensing receptor protein and is widely expressed in the parathyroid gland, kidneys, and intestines and regulated blood levels of calcium [51], whereas the other calcium SNPs are near to loci that are not known to have such a direct effect on calcium levels and may therefore be more prone to pleiotropy. The leave-one-out analyses, together with the Wald ratio result for *CASR* rs1801725 alone and the MR-Egger result, all suggest that our main IVW results might be biased by masking horizontal pleiotropy and that there is a modest positive causal effect of calcium on BW. Possible sources of masking pleiotropy could be alterations to glucose levels, as one of the genetic instruments (rs780094) is associated with fasting glucose, which is known to influence BW [52]. However, although these sensitivity analyses suggest a possible modest positive causal effect of maternal circulating calcium on BW, they have limited statistical power, and therefore they are imprecisely estimated with wide CIs. Although we were able to show most of the 25(OH)D SNPs related to pregnancy 25(OH)D, we were not able to assess this for maternal gestational calcium. If the calcium SNPs have weaker associations with circulating calcium levels in pregnancy (than they do in the GWAS of men [25]), this might bias towards the null any real effect of maternal pregnancy calcium on BW in our analyses. During pregnancy, maternal circulating levels of calcium increase in order to support healthy skeletal development. This is achieved through increasing absorption from the intestines and through bone resorption in the mothers, but this process is, at least partially, under the control of the fetus, which increases the secretion of parathyroid hormone-related protein in response to low fetal plasma calcium [53]. Thus, fetal genetic variants might be important genetic instruments for MR analyses of maternal circulating gestational calcium’s effect on fetal skeletal development and hence BW.

With respect to the difference between MR and instrumental variable analyses in RCT results, we might expect to see weaker effects from instrumental variable analyses in RCTs than from MR analyses, as the former only tests differences in exposure from the time of randomisation, whereas MR tests differences over most of life (and hence across all pregnancy trimesters). On the other hand, for in utero exposures, timing rather than duration of exposure might be important [20]. Though the fetus acquires all of its calcium from the mother throughout pregnancy, some evidence suggests that 80% of calcium in a neonate’s bones is absorbed during the third trimester (possibly because of an increase in maternal 1,25-dihydroxyvitamin D levels in the third trimester) [54], which would mean that supplementation starting earlier in pregnancy and continuing through to delivery might be necessary to ensure adequate levels for bone development throughout the third trimester (and hence an effect on BW). Five of the six RCTs that we included in our analyses would fit with this, as supplementation began at or before 23 weeks of gestation and continued until delivery (the one study that started supplementation in the third trimester—28 to 31 weeks—only included 32 participants). Overall, we might then expect the RCT and MR results to be similar. However, the results from the instrumental variable analyses in RCTs might be biased because of the inclusion of only six calcium supplementation RCTs, with the two largest ones having important sources of bias (S9 Table). Furthermore, whereas our MR analyses are largely in European-origin populations, the RCTs were predominantly in non-European populations, in particular Latin American populations, which may explain some of the differences.

### Study strengths and limitations

To the best of our knowledge, this is the first study to compare results from MR and instrumental variable analyses applied to RCTs to investigate the effect of maternal circulating calcium levels on offspring BW. The GWAS that we used for genetic instrument–exposure associations (sample 1) in our two-sample MR did not provide the percentage variation in calcium that all seven SNPs explained. However, based on a previous study that provided the R^2^ for one of these seven SNPs (rs1801725) [55], we know that our instrument with all seven SNPs will explain at least 2% of the variation in circulating calcium. We have considerably increased the sample size of our previous MR study of the effect of maternal circulating 25(OH)D on offspring BW [6]. These additions have increased the strength of our instrument, with the R^2^ in our study suggesting the genetic variants explained approximately 3% of variation in circulating 25(OH)D compared with <1% of the variation explained by the two SNPs used in the previous MR study [6]. We have explored the validity of our genetic instrumental variables using multiple sensitivity analyses and compared those results with instrumental variable analyses applied to RCTs, in which summary results from the RCTs were extracted independently by two people and checked for consistency with a third.

Key potential limitations include the low response to UKB and maternal report of first child’s BW many years after their birth. Recent research suggests that a highly select cohort (as in the case of UKB with a 5% response [56]) can result in selection bias in genetic or MR analyses [57,58]. Self-report of BW and the rounding to 1 pound (about 0.454 kg) may have introduced error in the BW measure in UKB, but this would be random with respect to genotype and expected to bias results towards the null. The somewhat lower mean BW in UKB participants than in ALSPAC/EFSOCH is likely to reflect secular trends of increasing birth size over time because of the fact that they reported the weight of first-born children only, whereas in ALSPAC/EFSOCH index children are from any pregnancy. The ALSPAC/EFSOCH MR estimates were stronger than the UKB estimates, though with very wide CIs reflecting their smaller sample size, and there is no evidence that the results were statistically inconsistent with those from UKB. These two cohorts were used in sensitivity analyses to explore whether the main MR analyses might be biased by a path from maternal genotype via fetal genotype to their BW. We have shown this is not the case. However, adjustment for fetal genotype could introduce a spurious association between maternal and paternal genotype [44], and if fathers’ circulating 25(OH)D or calcium influenced offspring BW, this could bias our results. As it is unlikely that fathers’ circulating levels of 25(OH)D or calcium could directly influence fetal growth and offspring BW, we feel our offspring-adjusted results are unlikely to be biased. This is also supported by the lack of any effect of own 25(OH)D or calcium on own BW in MR sensitivity analyses in UKB.

In UKB, the 25(OH)D genetic risk score was negatively associated with height, and as it is possible that greater maternal height results in greater BW, independently of the offspring genotype [59], this could mask any true positive effect of 25(OH)D on BW. We also found that the calcium genetic instrument was associated with markers of socioeconomic position (area deprivation and education), which could confound the MR estimated effect of calcium on BW. However, results of multivariable MR adjusting the maternal 25(OH)D effect on BW for maternal height and the maternal calcium effect on BW (partially) for maternal education were consistent with the unadjusted results, suggesting that our results were not importantly confounded.

Horizontal pleiotropy is a key source of bias for MR studies. For the effects of maternal circulating 25(OH)D on BW, the consistency of results across all of our MR analyses suggests that this has not been a key source of bias for that exposure. We did find evidence of potential horizontal pleiotropy with maternal calcium effects and as discussed above and acknowledge that further studies are required to explore that effect. We have assumed that genetic variants that relate to 25(OH)D/calcium in (nonpregnant) women and men have the same magnitudes of association with these exposures in pregnant women. We were unable to test this for calcium but did show that five out of the seven variants related similarly to circulating 25(OH)D during pregnancy as in the GWAS. Removal of either of the two that did not associate as strongly to 25(OH)D in pregnancy in our leave-one-out analyses was consistent with the main results and all other leave-one-out analyses. Although we aimed to use randomisation to use supplementation with vitamin D or calcium as an instrumental variable for RCTs, some studies did not provide results from intention-to-treat analyses. Therefore, results from these studies could be biased by noncompliance. The aim of triangulation is to compare results from different methods that have different key sources of bias. Although both approaches that we have used here rely on instrumental variables and have the same underlying assumptions, the sources of violation of these assumptions differ between MR and instrumental variables applied to RCTs. For MR, horizontal pleiotropy is the key source of bias, whereas for instrumental variables applied to RCT, it is lack of concealed random allocation (which can introduce bias) and not using intention-to-treat analysis (which can produce a path from the instrument to outcome and/or introduce confounding). The fact that there was no marked difference between our MR and RCT analyses strengthens confidence in the findings for 25(OH)D. However, as discussed above, the different results for calcium need further exploration. In both our two-sample MR and instrumental variable analyses applied to RCTs, we have used summary data and are unable to explore possible nonlinear effects, though observational studies do not suggest that these are present. Similarly, we cannot explore effect modification, but we are not aware of any evidence that the effects that we have looked at do differ by other characteristics. Our MR analyses are in white European populations and may not generalise to other groups, such as those with different levels of skin pigmentation and exposure to sunshine. However, most of the RCTs for vitamin D and calcium supplementation are in non-Europeans. In particular, most vitamin D RCTs were in Middle Eastern or South Asian populations, and a recent RCT in Bangladesh, which was not included in the systematic review that we used in this paper for the RCT analyses, found that vitamin D supplementation during pregnancy made no difference to BW [60]. Thus, even in a low-income, dark-skinned population, maternal circulating 25(OH)D may not be an important factor in BW.

In conclusion, our results suggest that maternal circulating 25(OH)D does not have a clinically important effect on BW, so pregnancy supplementation with vitamin D is unlikely to affect mean BW. Higher maternal circulating calcium may increase BW, but further research is required to clarify this, including larger samples for undertaking pleiotropy ‘adjusted’ MR analyses and larger, better-conducted RCTs of calcium supplementation.

## Supporting information

S1 Checklist (DOCX)

S1 Text Study descriptions. (DOCX)

S2 Text Selecting of participants of white European ancestry. (DOCX)

S3 Text Selecting participants for own birth weight analyses in UK Biobank. (DOCX)

S4 Text Measuring 25(OH)D during gestation in mothers in the ALSPAC cohort. ALSPAC, Avon Longitudinal Study of Parents and Children.
(DOCX)

S5 Text Sensitivity analysis to explore additional sources of invalid instruments. (DOCX)

S1 Table Characteristics of the genome-wide association studies of 25(OH)D. (PDF)

S2 Table Details of SNPs used in our mendelian randomisation analyses. SNP, single-nucleotide polymorphism.
(PDF)

S3 Table Studies used to calculate the RCT instrumental variable effect of 25(OH)D on birth weight. RCT, randomised controlled trial.
(PDF)

S4 Table Studies used to calculate the RCT instrumental variable effect of calcium on birth weight. RCT, randomised controlled trial.
(PDF)

S5 Table SNP effects on first child’s birth weight in all studies. SNP, single-nucleotide polymorphism.
(PDF)

S6 Table SNP effects on own birth weight in UK Biobank (N = 215,444). SNP, single-nucleotide polymorphism.
(PDF)

S7 Table SNP effects on fetal adjusted birth weight in ALSPAC and EFSOCH. ALSPAC, Avon Longitudinal Study of Parents and Children; EFSOCH, Exeter Family Study of Childhood Health; SNP, single-nucleotide polymorphism.
(PDF)

S8 Table Risk of bias in studies included in IV of RCT analyses of vitamin D supplementation. IV, instrumental variable; RCT, randomised controlled trial.
(PDF)

S9 Table Risk of bias in studies included in IV of RCT analyses of calcium supplementation. IV, instrumental variable; RCT, randomised controlled trial.
(PDF)

S10 Table Associations between weighted allele scores and potential confounders. (PDF)

S11 Table Multivariable MR for 25(OH)D and calcium effect on birth weight in UK Biobank (adjusting for height effects). MR, mendelian randomisation.
(PDF)

S1 Fig Flow diagram of participant inclusion for ALSPAC and EFSOCH. ALSPAC, Avon Longitudinal Study of Parents and Children; EFSOCH, Exeter Family Study of Childhood Health.
(PDF)

S2 Fig Flow diagram for participant inclusion in UK Biobank. (PDF)

S3 Fig Flow diagram for inclusion of trials in the instrumental variables applied to RCTs. RCT, randomised controlled trial.
(PDF)

S4 Fig Leave-one-out analysis for effect of maternal gestational circulating 25(OH)D on birth weight mendelian randomisation Wald ratio estimate in UK Biobank. (PDF)

S5 Fig Leave-one-out analysis for effect of 25(OH)D on birth weight RCT instrumental variable Wald ratio estimate. RCT, randomised controlled trial.
(PDF)

S6 Fig Mendelian randomisation effect estimates for maternal 25(OH)D synthesis and metabolism on birth weight in UK Biobank. (PDF)

S7 Fig Leave-one-out analysis for effect of maternal gestational circulating calcium on birth weight mendelian randomisation Wald ratio estimate in UK Biobank. (PDF)

S8 Fig Leave-one-out analysis for effect of maternal gestational circulating calcium RCT instrumental variable Wald ratio estimate. RCT, randomised controlled trial.
(PDF)

## Abbreviations

ALSPAC: Avon Longitudinal Study of Parents and Children
*AMDHD1*: Amidohydrolase Domain Containing 1
BMD: bone mineral density
BMI: body mass index
BW: birth weight
*CASR*: calcium-sensing receptor
*DHCR7*: 7-Dehydrocholesterol reductase
EFSOCH: Exeter Family Study of Childhood Health
GRM: genetic relationship matrix
GWA: genome-wide association
GWAS: genome-wide association studies
HWE: Hardy Weinberg equilibrium
IVW: inverse variance–weighted
MR: mendelian randomisation
RCT: randomised controlled trial
SNP: single nucleotide polymorphism
STROBE: Strengthening the Reporting of Observational Studies in Epidemiology
TDI: Townsend deprivation index
UKB: UK Biobank
