# Supplementary material for: Association of maternal circulating 25(OH)D and calcium with birth weight: A mendelian randomisation analysis
Source: PLoS Med. 2019 Jun 18;16(6):e1002828. doi: 10.1371/journal.pmed.1002828 (PMC6581250; doi:10.1371/journal.pmed.1002828)
Supplement: S1 Checklist — (DOCX) [file pmed.1002828.s001.docx]

STROBE Statement—Checklist of items that should be included in reports of ***cohort studies***

|  | Item No | Recommendation |
| --- | --- | --- |
| **Title and abstract** | 1 | 1. Indicate the study’s design with a commonly used term in the title or the abstract   **Title Page** |
|  |  | 1. Provide in the abstract an informative and balanced summary of what was done and what was found   **Abstract: Methods and Findings** |
| Introduction | | |
| Background/rationale | 2 | Explain the scientific background and rationale for the investigation being reported  **Introduction: paragraphs 1, 2 and 3** |
| Objectives | 3 | State specific objectives, including any prespecified hypotheses  **Introduction: paragraph 3** |
| Methods | | |
| Study design | 4 | Present key elements of study design early in the paper  **Methods: sections under subheadings: Mendelian Randomisation; Statistical Analysis; and Instrumental variable analysis applied to RCTs. Also summarised in Figure 1.** |
| Setting | 5 | Describe the setting, locations, and relevant dates, including periods of recruitment, exposure, follow-up, and data collection  **Supplementary Text: S1 Text: Study Descriptions, UK Biobank, ALSPAC and EFSOCH** |
| Participants | 6 | 1. Give the eligibility criteria, and the sources and methods of selection of participants. Describe methods of follow-up   **Methods: sections under subheadings: Birth weight and serum 25(OH)D measurement, Genotyping and SNP selection, and summary data for SNP-25(OH)D and SNP-calcium associations.**  **Supplementary Text: S2 Text and S3 Text: Selecting of participants of White European ancestry and Selecting participants for own birth weight analyses in UK Biobank** |
|  |  | 1. For matched studies, give matching criteria and number of exposed and unexposed   **Not applicable** |
| Variables | 7 | Clearly define all outcomes, exposures, predictors, potential confounders, and effect modifiers. Give diagnostic criteria, if applicable  **Methods: sections under subheadings: Mendelian Randomisation, Birth weight and serum 25(OH)D measurement, Genotyping, SNP selection, and summary data for SNP-25(OH)D and SNP-calcium associations, Exploring possible violations of MR assumptions and Instrumental variable analysis applied to RCTs.**  **Supplementary Text: S2 Text, S3 Text and S5 text: Selecting of participants of White European ancestry, Selecting participants for own birth weight analyses in UK Biobank and Checking associations of SNPs with observed confounders of gestational 25(OH)D/calcium birth weight associations.** |
| Data sources/ measurement | 8* | For each variable of interest, give sources of data and details of methods of assessment (measurement). Describe comparability of assessment methods if there is more than one group  **Method: sections under subheadings: Mendelian Randomisation, Birth weight and serum 25(OH)D measurement, Genotyping, SNP selection, and summary data for SNP-25(OH)D and SNP-calcium associations, Exploring possible violations of MR assumptions and Instrumental variable analysis applied to RCTs.**  **Supplementary Text: S2 Text, S3 Text and S5 text: Selecting of participants of White European ancestry, Selecting participants for own birth weight analyses in UK Biobank and Checking associations of SNPs with observed confounders of gestational 25(OH)D/calcium birth weight associations.** |
| Bias | 9 | Describe any efforts to address potential sources of bias  **Methods: sections under subheadings: Statistical Analyses and Exploring possible violations of MR assumptions.**  **Supplementary Text: S5 Text: Sensitivity analysis to explore additional sources of invalid instruments.** |
| Study size | 10 | Explain how the study size was arrived at  **Methods: sections under subheadings: Birth weight and serum 25(OH)D measurement and Instrumental variable analysis applied to RCTs.**  **Supplementary Text: S3 Text: Selecting participants for own birth weight analyses in UK Biobank.** |
| Quantitative variables | 11 | Explain how quantitative variables were handled in the analyses. If applicable, describe which groupings were chosen and why  **Methods: sections under subheadings: Statistical Analyses, Exploring possible violations of MR assumptions and Instrumental variable analysis applied to RCTs.**  **Supplementary Text: S5 Text: Sensitivity analysis to explore additional sources of invalid instruments.** |
| Statistical methods | 12 | 1. Describe all statistical methods, including those used to control for confounding   **Methods: sections under subheadings: Statistical Analyses, Exploring possible violations of MR assumptions and Instrumental variable analysis applied to RCTs.**  **Supplementary Text: S5 Text: Sensitivity analysis to explore additional sources of invalid instruments.** |
|  |  | 1. Describe any methods used to examine subgroups and interactions   **Methods: Exploring possible violations of MR assumptions, paragraph 2.** |
|  |  | (*c*) Explain how missing data were addressed  **Methods: sections under subheadings: Birth weight and serum 25(OH)D measurement, Genotyping and SNP selection, and summary data for SNP-25(OH)D and SNP-calcium associations.**  **Supplementary Text: S2 Text and S3 Text: Selecting of participants of White European ancestry and Selecting participants for own birth weight analyses in UK Biobank** |
|  |  | 1. If applicable, explain how loss to follow-up was addressed   **Not applicable** |
|  |  | 1. Describe any sensitivity analyses   **Methods: sections under subheadings: Exploring possible violations of MR assumptions and Instrumental variable analysis applied to RCTs.**  **Supplementary Text: S5 Text: Sensitivity analysis to explore additional sources of invalid instruments.** |
| Results | | |
| Participants | 13* | 1. Report numbers of individuals at each stage of study—eg numbers potentially eligible, examined for eligibility, confirmed eligible, included in the study, completing follow-up, and analysed   **Methods: Figure 1**  **Methods: Table 1**  **Results, paragraph 1.**  **Supplementary tables 3 and 4.**  **Supplementary figures 1, 2 and 3.** |
|  |  | 1. Give reasons for non-participation at each stage   **Supplementary figures 1, 2 and 3.** |
|  |  | 1. Consider use of a flow diagram   **Methods: Figure 1.**  **Supplementary figures 1, 2 and 3.** |
| Descriptive data | 14* | 1. Give characteristics of study participants (eg demographic, clinical, social) and information on exposures and potential confounders   **Methods: Table 1** |
|  |  | 1. Indicate number of participants with missing data for each variable of interest   **Supplementary figures 1, 2 and 3.** |
|  |  | 1. Summarise follow-up time (eg, average and total amount)   **Not applicable** |
| Outcome data | 15* | Report numbers of outcome events or summary measures over time  **Supplementary tables 3, 4, 5, 6, 7, 8 and 9.** |
| Main results | 16 | 1. Give unadjusted estimates and, if applicable, confounder-adjusted estimates and their precision (eg, 95% confidence interval). Make clear which confounders were adjusted for and why they were included   **Results: Figures 2 and 3** |
|  |  | 1. Report category boundaries when continuous variables were categorized   **Methods: Table 1** |
|  |  | 1. If relevant, consider translating estimates of relative risk into absolute risk for a meaningful time period   **Not applicable** |
| Other analyses | 17 | Report other analyses done—eg analyses of subgroups and interactions, and sensitivity analyses  **Results: Figures 2 and 3**  **Supplementary tables 10 and 11.**  **Supplementary Figures 4, 5, 6, 7 and 8.** |
| Discussion | | |
| Key results | 18 | Summarise key results with reference to study objectives  **Discussion: paragraphs 1-4** |
| Limitations | 19 | Discuss limitations of the study, taking into account sources of potential bias or imprecision. Discuss both direction and magnitude of any potential bias  **Discussion: in section with subheading: Study Strengths and Limitations** |
| Interpretation | 20 | Give a cautious overall interpretation of results considering objectives, limitations, multiplicity of analyses, results from similar studies, and other relevant evidence  **Discussion: Study Strengths and Limitations, paragraph 3** |
| Generalisability | 21 | Discuss the generalisability (external validity) of the study results  **Discussion: paragraphs 1-4 and Study Strengths and Limitations** |
| Other information | | |
| Funding | 22 | Give the source of funding and the role of the funders for the present study and, if applicable, for the original study on which the present article is based  **Funding.** |

*Give information separately for exposed and unexposed groups.

**Note:** An Explanation and Elaboration article discusses each checklist item and gives methodological background and published examples of transparent reporting. The STROBE checklist is best used in conjunction with this article (freely available on the Web sites of PLoS Medicine at http://www.plosmedicine.org/, Annals of Internal Medicine at http://www.annals.org/, and Epidemiology at http://www.epidem.com/). Information on the STROBE Initiative is available at http://www.strobe-statement.org.
