# Supplementary material for: Association of maternal circulating 25(OH)D and calcium with birth weight: A mendelian randomisation analysis
Source: PLoS Med. 2019 Jun 18;16(6):e1002828. doi: 10.1371/journal.pmed.1002828 (PMC6581250; doi:10.1371/journal.pmed.1002828)
Supplement: S1 Text — (DOCX) [file pmed.1002828.s002.docx]

**S1 Text: Study Descriptions**

UK Biobank

Between 2006 and 2010, patients were recruited from the NHS patient registers and contacted if they lived in close proximity to one of 22 assessment centres in England, Scotland and Wales. Detailed medical data was collected on 502,655 participants, aged between 40 and 69 at recruitment [1]. A total of 190,406 women in the UK Biobank cohort who had reported their first child’s birth weight (BW) were included in the primary analyses of this paper. All participants provided written informed consent, including for their collected data to be used by international scientists. UK Biobank has approval from the North West Multi-centre Research Ethics Committee (MREC), which covers the UK. UK Biobank’s research ethics committee and Human Tissue Authority research tissue bank approvals mean that researchers wishing to use the resource do not need separate ethics approval.

ALSPAC

Women expecting a live birth between the 1st of April 1991 and 31st of December 1992 whilst living in Avon, UK were invited to take part in the study. Initially 14,541 pregnancies were recruited, which resulted in 14,676 fetuses, 14,062 live births and 13,988 children alive after one year, with additional children being recruited later [2,3]. Please note that the study website contains details of all the data that is available through a fully searchable data dictionary and variable search tool [4]. Mothers provided written informed consent and ethical approval for the study was obtained from the ALSPAC Ethics and Law Committee and the Local Research Ethics Committees.

EFSOCH

Between 2000 and 2004, pregnant women from a postcode defined region of Exeter, UK and their partners were recruited via the Exeter Maternity Unit database. A total of 1,017 families (98% white European) were recruited [5], from which a total of 993 live births were included in the primary analyses of this paper. All mothers and fathers gave informed consent and ethical approval was obtained from the local review committee.

**References**

1. Hewitt J, Walters M, Padmanabhan S, Dawson J. Cohort profile of the UK Biobank: diagnosis and characteristics of cerebrovascular disease. BMJ Open. 2016;6(3).

2. Boyd A, Golding J, Macleod J, Lawlor DA, Fraser A, Henderson J, et al. Cohort Profile: The ‘Children of the 90s’—the index offspring of the Avon Longitudinal Study of Parents and Children. International Journal of Epidemiology. 2013;42(1):111-27. doi: 10.1093/ije/dys064.

3. Fraser A, Macdonald-Wallis C, Tilling K, Boyd A, Golding J, Davey Smith G, et al. Cohort Profile: The Avon Longitudinal Study of Parents and Children: ALSPAC mothers cohort. International Journal of Epidemiology. 2013;42(1):97-110. doi: 10.1093/ije/dys066.

4. Explore data and samples: University of Bristol; 2002-2017 [cited 2018 16th of August]. Available from: <http://www.bristol.ac.uk/alspac/researchers/our-data/>.(last accessed 2019 1st of March)

5. Knight B, Shields BM, Hattersley AT. The Exeter Family Study of Childhood Health (EFSOCH): study protocol and methodology. Paediatric and Perinatal Epidemiology. 2006;20(2):172-9. doi: 10.1111/j.1365-3016.2006.00701.x.
