# Supplementary material for: Association of maternal circulating 25(OH)D and calcium with birth weight: A mendelian randomisation analysis
Source: PLoS Med. 2019 Jun 18;16(6):e1002828. doi: 10.1371/journal.pmed.1002828 (PMC6581250; doi:10.1371/journal.pmed.1002828)
Supplement: S2 Text — (DOCX) [file pmed.1002828.s003.docx]

**S2 Text: Selecting of participants of White European ancestry**

In UK Biobank, we defined a subset of “white European” ancestry participants so that only those of this ethnic background were included in our analyses. To do this, we generated ancestry principal components (PCs) in the 1000 genomes samples. The UKB samples were then projected into this PC space using the SNP loadings obtained from the principal components analysis using the 1000 genomes samples. The UK Biobank participants’ ancestry was classified using K-means clustering centred on the 3 main 1000 genomes populations (European, African, South Asian). Those clustering with the European cluster were classified as having European ancestry. The UK Biobank participants were asked to report their ethnic background. Only those reporting as either “British”, “Irish”, “White” or “Any other white background” were included in the clustering analysis.

For ALSPAC, we also used PCs in the 1000 genomes sample to separate out white Europeans in the genotyped individuals (see above).

EFSOCH only included participants of white British origin (defined using PCs) for analyses[2]. Nonetheless, principal component analysis was performed to assess ancestry of the sample using flashPCA [2]. Outliers were defined as >4.56 SD from the cluster mean (defined using 1000 Genomes European data as the reference) and excluded (n=21 individuals [0.76%])

**References**

1. Knight B, Shields BM, Hattersley AT. The Exeter Family Study of Childhood Health (EFSOCH): study protocol and methodology. Paediatric and Perinatal Epidemiology. 2006;20(2):172-9. doi: 10.1111/j.1365-3016.2006.00701.x.

2. Abraham G, Inouye M. Fast Principal Component Analysis of Large-Scale Genome-Wide Data. PLOS ONE. 2014;9(4):e93766. doi: 10.1371/journal.pone.0093766.
