# Supplementary material for: Association of maternal circulating 25(OH)D and calcium with birth weight: A mendelian randomisation analysis
Source: PLoS Med. 2019 Jun 18;16(6):e1002828. doi: 10.1371/journal.pmed.1002828 (PMC6581250; doi:10.1371/journal.pmed.1002828)
Supplement: S3 Text — (DOCX) [file pmed.1002828.s004.docx]

**S3 Text: Selecting participants for own birth weight analyses in UK Biobank**

A total of 280,315 participants reported their own BW in kilograms at either the baseline visit or at least one of the follow-up visits. Participants reporting being part of a multiple birth were excluded from our analyses (N=10,057). For participants reporting BW at more than one visit (N=11,629), the average across the reported BWs were used, and if the largest difference between any 2 time points was >1kg, they were excluded (N=80). Data on gestational duration were not available. However, in order to exclude likely pre-term births, participants with BW values <2.5kg were excluded. We also excluded those with a BW >4.5kg as these are likely to be reporting errors or extreme outliers (total number excluded because of <2.5kg or >4.5kg BW =37,691). Participants’ own BW was regressed against year of birth and assessment centre location. Residuals from that regression model were then used in all analyses with values converted to standard deviation units for analysis.
