# Supplementary material for: Association of maternal circulating 25(OH)D and calcium with birth weight: A mendelian randomisation analysis
Source: PLoS Med. 2019 Jun 18;16(6):e1002828. doi: 10.1371/journal.pmed.1002828 (PMC6581250; doi:10.1371/journal.pmed.1002828)
Supplement: S4 Text — ALSPAC, Avon Longitudinal Study of Parents and Children. (DOCX) [file pmed.1002828.s005.docx]

**S4 Text:** **Measuring 25(OH)D during gestation in mothers in the ALSPAC cohort**

Serum samples could be from any gestational age and some women had more than one measure of 25(OH)D in pregnancy. The dates of blood sampling were obtained from medical records and used to calculate gestational age at the time of maternal 25(OH)D measurement and adjustment for seasonality. Sine-cosine regression was used to adjust for seasonality, with all measurements (including repeat measures within some women) used in these analyses to obtain a predicted seasonal adjusted mid-third trimester measurements in all women with at least one pregnancy 25(OH)D measure, as described previously[1].

**References**

1. Lawlor DA, Wills AK, Fraser A, Sayers A, Fraser WD, Tobias JH. Association of maternal vitamin D status during pregnancy with bone-mineral content in offspring: a prospective cohort study. Lancet. 2013;381(9884):2176-83. doi: 10.1016/S0140-6736(12)62203-X.
