# Supplementary material for: Association of maternal circulating 25(OH)D and calcium with birth weight: A mendelian randomisation analysis
Source: PLoS Med. 2019 Jun 18;16(6):e1002828. doi: 10.1371/journal.pmed.1002828 (PMC6581250; doi:10.1371/journal.pmed.1002828)
Supplement: S5 Text — (DOCX) [file pmed.1002828.s006.docx]

**S5 Text: Sensitivity analysis to explore additional sources of invalid instruments**

MR-Egger

Like IVW, MR-Egger uses linear regression of the SNP associations with birth weight against the SNP associations with 25(OH)D or calcium, but MR-Egger does not force the intercept through zero, thus relaxing the assumption that the SNP influences birth weight only through the 25(OH)D or calcium (see Table 1 of main paper)[1]. If a non-zero intercept is observed, this indicates that there may be bias in the fixed effect pooled Wald Ratios and/or IVW instrumental variable estimates due to horizontal pleiotropy. Whilst relaxing the no horizontal pleiotropy assumption and providing an estimate that takes account of non-symmetrical pleiotropy (the slope value), MR-Egger introduces an additional assumption - the Instrument Strength Independent of Direct Effect (INSIDE) assumption. INSIDE assumes that the association of the genetic instrument with the exposure is not correlated with the association of the genetic instrument with the outcome (i.e. the association with outcome that is not via the exposure of interest). In relation to this study the INSIDE assumption is likely to be violated via offspring genotype because of the association of maternal genotype to risk factor and to her offspring genotype[2], and so MR-Egger is unlikely to be a useful approach for testing this source of bias. We use adjustment for offspring genotype to test this (see methods in main paper) and used MR-Egger as a sensitivity analysis to explore possible violation of the exclusion restriction criteria via maternal genetic horizontal pleiotropy. For our MR-Egger analyses we estimated the standard error using a fixed effects model and confidence intervals using a t-distribution.

Weighted-Median Analysis

With weighted-median analysis, the weighted-median instrumental variable of all the SNPs is taken as the causative effect, with each SNP being weighted by its effect on the exposure, thus reducing the effect of single weak instruments (see Table 1 in main paper)[3]. This method also relaxes the assumption of there being no bias due to asymmetrical horizontal pleiotropy but it assumes that no more than 50% of the combined SNPs weight is from invalid instruments. This approach will be biased if there is a single horizontal pleiotropic SNP with 50% of the weight or multiple pleiotropic SNPs, each with less than 50% of the weight, but that together are 50% or more of the weight. As with MR-Egger this is likely to be violated by offspring genotype as 50% of maternal alleles will be transferred to the fetus; our fetal genotype adjusted results are the key way of testing for bias via that route[2]. The weighted median analyses were as a sensitivity analysis to explore possible violation of the exclusion restriction criteria via maternal genetic horizontal pleiotropy.

Checking associations of SNPs with observed confounders of gestational 25(OH)D/calcium birth weight associations

To explore the possible association of SNPs with observed confounders, we calculated two weighted allele scores (WAS) from the instrumental variable SNPs for 25(OH)D/calcium and determined the per allele association of these WAS with each confounder. Each SNP was weighted by the magnitude of its effect on the exposure as reported in the original GWAS. The potential confounders we calculated WAS for were; mothers pre-pregnancy BMI, height and smoking (all three in UK Biobank, ALSPAC and EFSOCH), mothers systolic blood pressure and educational attainment (UK Biobank and ALSPAC only), mothers Townsend area of residence deprivation index[4] (UK Biobank and EFSOCH only) and mothers adherence to a Western Diet (UK Biobank only).

Multivariable MR

To adjust for maternal height in the MR analyses of 25(OH)D effects on BW we used genetic instruments (N = 696 SNPs) for from the most recent GWAS of height that had reached genome-wide significance and replicated[5]. To adjust for maternal education in the multivariable MR of the effect of calcium on BW we aimed to use SNPs that were genome-wide significant and replicated from the most recent GWAS of completed years of education that were genome wide significant and replicated[6]. In both analyses we used the IVW method for the multivariable MR analyses this requires summary data on all of the: exposure SNP associations with exposure, outcome and confounder and confounder SNP associations with confounder outcome and exposure. In the analyses of calcium we were unable to do this because data were only provided for the genome-wide significant hits and not the whole genome which meant that we could not find confounder (maternal education) SNP associations with calcium for all of the education hits. We therefore did a partial multivariable MR to adjust calcium-BW effects for maternal education.

**References**

1. Bowden J, Davey Smith G, Burgess S. Mendelian randomization with invalid instruments: effect estimation and bias detection through Egger regression. International Journal of Epidemiology. 2015;44(2):512-25. doi: 10.1093/ije/dyv080.

2. Lawlor D, Richmond R, Warrington N, McMahon G, Davey Smith G, Bowden J, et al. Using Mendelian randomization to determine causal effects of maternal pregnancy (intrauterine) exposures on offspring outcomes: Sources of bias and methods for assessing them. Wellcome open research. 2017;2:11-. doi: 10.12688/wellcomeopenres.10567.1.

3. Bowden J, Davey Smith G, Haycock PC, Burgess S. Consistent Estimation in Mendelian Randomization with Some Invalid Instruments Using a Weighted Median Estimator. Genetic Epidemiology. 2016;40(4):304-14. doi: 10.1002/gepi.21965.

4. Townsend P, Phillimore P, Beattie A. Health and Deprivation: Inequality and the North. London: Routledge; 1988.

5. Wood AR, Esko T, Yang J, Vedantam S, Pers TH, Gustafsson S, et al. Defining the role of common variation in the genomic and biological architecture of adult human height. Nature genetics. 2014;46:1173. doi: 10.1038/ng.3097.

6. Okbay A, Beauchamp JP, Fontana MA, Lee JJ, Pers TH, Rietveld CA, et al. Genome-wide association study identifies 74 loci associated with educational attainment. Nature. 2016;533:539. doi: 10.1038/nature17671.
