# Supplementary material for: Association of maternal circulating 25(OH)D and calcium with birth weight: A mendelian randomisation analysis
Source: PLoS Med. 2019 Jun 18;16(6):e1002828. doi: 10.1371/journal.pmed.1002828 (PMC6581250; doi:10.1371/journal.pmed.1002828)
Supplement: S1 Table — (PDF) [file pmed.1002828.s007.pdf]

S1 Table: Characteristics of the genome-wide association studies of 25(OH)D

Study	Trait	Population	No of Discovery cohorts	No of Discovery participants	No of Recovery cohorts	No of Recovery participants	N (total)	N(%) of participants that were female
Manousaki et al 2017[1]	25[OH]D	European	2	2,619	17	39,655	42,274	39.5
Jiang et al 2018[2]	25[OH]D	European	31	79,366	2	42,757	122,123	NA
O'Seaghdha et al 2013[3]	Calcium	European	19	39,400	11	21,875	61,275	0

NA: data on proportion of males and females not provided

References

1. Manousaki D, Dudding T, Haworth S, Hsu Y-H, Liu C-T, Medina-Gómez C, et al. Low-Frequency Synonymous Coding Variation in CYP2R1 Has Large Effects on Vitamin D Levels and Risk of Multiple Sclerosis. The American Journal of Human Genetics. 2017;101(2):227-38. doi: https://doi.org/10.1016/j.ajhg.2017.06.014.
2. Jiang X, O'Reilly PF, Aschard H, Hsu YH, Richards JB, Dupuis J, et al. Genome-wide association study in 79,366 European-ancestry individuals informs the genetic architecture of 25-hydroxyvitamin D levels. Nature communications. 2018;9(1):260. Epub 2018/01/19. doi: 10.1038/s41467-017-02662-2.
3. O'Seaghdha CM, Wu H, Yang Q, Kapur K, Guessous I, Zuber AM, et al. Meta-Analysis of Genome-Wide Association Studies Identifies Six New Loci for Serum Calcium Concentrations. PLOS Genetics. 2013;9(9):e1003796. doi: 10.1371/journal.pgen.1003796.
