# Supplementary material for: Association of maternal circulating 25(OH)D and calcium with birth weight: A mendelian randomisation analysis
Source: PLoS Med. 2019 Jun 18;16(6):e1002828. doi: 10.1371/journal.pmed.1002828 (PMC6581250; doi:10.1371/journal.pmed.1002828)
Supplement: S2 Table — SNP, single-nucleotide polymorphism. (PDF) [file pmed.1002828.s008.pdf]

S2 Table: Details of SNPs used in our Mendelian Randomisation analyses

Trait	SNP	Nearest/Nearby gene	Trait raising allele	Trait lowering allele	Trait raising allele frequency (GWAS reported)	Difference in mean 25(OH)D or calcium (95% CI) per allele	Units of change in 25(OH)D or calcium per allele and GWAS used to obtain these results
Vitamin D (synthesis)	rs10741657	*CYP2R1*	A	G	0.4	0.031 (0.027 to 0.035)	Log nmol/l (Jiang et al (2018) Nature Communications)[1]
Vitamin D (synthesis)	rs117913124	*CYP2R1*	G	A	0.975	0.21 (0.19 to 0.23)*	Log nmol/l (Manousaki et al (2017) The American Journal of Human Genetics) [2]
Vitamin D (synthesis)	rs12785878	*DHCR7*	T	G	0.75	0.036 (0.032 to 0.04)	Log nmol/l (Jiang et al (2018) Nature Communications)[1]
Vitamin D (metabolism)	rs3755967	*GC*	C	T	0.72	0.089 (0.084 to 0.094)	Log nmol/l (Jiang et al (2018) Nature Communications)[1]
Vitamin D (metabolism)	rs17216707	*CYP24A1*	T	C	0.79	0.026 (0.021 to 0.031)	Log nmol/l (Jiang et al (2018) Nature Communications)[1]
Vitamin D	rs10745742	*AMDHD1*	T	C	0.41	0.017 (0.013 to 0.021)	Log nmol/l (Jiang et al (2018) Nature Communications)[1]

Vitamin D	rs8018720	*SEC23A*	G	C	0.27	0.017 (0.012 to 0.022)	Log nmol/l (Jiang et al (2018) Nature Communications)[1]
Calcium	rs1801725	*CASR*	T	G	0.15	0.071 (0.063 to 0.079)	Mg/dl (O'Seaghdha et al (2013) PLOS Genetics)[3]
Calcium	rs1550532	*DGKD*	C	G	0.31	0.018 (0.012 to 0.024)	Mg/dl (O'Seaghdha et al (2013) PLOS Genetics)[3]
Calcium	rs780094	*GCKR*	T	C	0.41	0.017 (0.011 to 0.023)	Mg/dl (O'Seaghdha et al (2013) PLOS Genetics)[3]
Calcium	rs10491003	*GATA3*	T	C	0.09	0.027 (0.017 to 0.037)	Mg/dl (O'Seaghdha et al (2013) PLOS Genetics)[3]
Calcium	rs7481584	*CARS*	G	A	0.71	0.018 (0.012 to 0.024)	Mg/dl (O'Seaghdha et al (2013) PLOS Genetics)[3]
Calcium	rs7336933	*DGKH; KIAA0564*	G	A	0.85	0.022 (0.014 to 0.03)	Mg/dl (O'Seaghdha et al (2013) PLOS Genetics)[3]
Calcium	rs1570669	*CYP24A1*	G	A	0.33	0.018 (0.012 to 0.024)	Mg/dl (O'Seaghdha et al (2013) PLOS Genetics)[3]

*The reported value in Manousaki et al[2] was 0.43 (0.39 to 0.47) standard deviations of 25(OH)D in log nmol/L, to get the value in log nmol/L we multiplied by 0.487, the standard deviation for 25(OH)D levels in ALSPAC in log nmol/L.

References

1. Jiang X, O'Reilly PF, Aschard H, Hsu YH, Richards JB, Dupuis J, et al. Genome-wide association study in 79,366 European-ancestry individuals informs the genetic architecture of 25-hydroxyvitamin D levels. Nature communications. 2018;9(1):260. Epub 2018/01/19. doi: 10.1038/s41467-017-02662-2.
2. Manousaki D, Dudding T, Haworth S, Hsu Y-H, Liu C-T, Medina-Gómez C, et al. Low-Frequency Synonymous Coding Variation in CYP2R1 Has Large Effects on Vitamin D Levels and Risk of Multiple Sclerosis. The American Journal of Human Genetics. 2017;101(2):227-38. doi: https://doi.org/10.1016/j.ajhg.2017.06.014.
3. O'Seaghdha CM, Wu H, Yang Q, Kapur K, Guessous I, Zuber AM, et al. Meta-Analysis of Genome-Wide Association Studies Identifies Six New Loci for Serum Calcium Concentrations. PLOS Genetics. 2013;9(9):e1003796. doi: 10.1371/journal.pgen.1003796.
