# Supplementary material for: Association of maternal circulating 25(OH)D and calcium with birth weight: A mendelian randomisation analysis
Source: PLoS Med. 2019 Jun 18;16(6):e1002828. doi: 10.1371/journal.pmed.1002828 (PMC6581250; doi:10.1371/journal.pmed.1002828)
Supplement: S3 Table — RCT, randomised controlled trial. (PDF) [file pmed.1002828.s009.pdf]

S3 Table: Studies used to calculate the RCT instrumental variable effect of 25(OH)D on birth weight

Studies	Number of participants in the study	Mean Difference in birth weight between supplementation and placebo group, in g	Mean Difference in 25(OH)D levels between supplementation and placebo group, in nmol/l
Mutlu (L) 2014[1]	59	24 (-379.48 to 427.48)	2.75 (-8.93 to 14.42)
Yu (H) 2009[2]	120	22 (-240.1 to 284.1)	5.67 (1.28 to 10.06)
Khan (2016)[3]	85	-180 (-488.76 to 128.76)	9.98 (2.46 to 17.51)
Vaziri (2016)[4]	127	-58 (-203.82 to 87.82)	15.03 (8.1 to 21.95)
Mallet (L) (1986)[5]	56	-90 (-339.46 to 159.46)	15.9 (11.75 to 20.05)
Mallet (H) (1986)[5]	50	-250 (-512.22 to 12.22)	16.6 (13.11 to 20.09)
Dawodu (L) (2013)[6]	129	91 (-102.45 to 284.45)	16.67 (5.13 to 28.22)
Yu (L) (2009)[2]	120	53 (-214.38 to 320.38)	18.67 (9.64 to 27.69)
Mutlu (H) (2014)[1]	60	-60 (-459.98 to 339.38)	18.97 (9.73 to 28.21)
Hollis (L) (2011)[7]	333	138.3 (-67.38 to 343.98)	19.4 (8.04 to 30.76)
Sahoo (L) (2016)[8]	39	110 (-278.99 to 498.99)	22.8 (8.21 to 37.39)
Thiele (2016)[9]	13	-102 (-638.05 to 434.05)	23.14 (15.41 to 30.87)
Mojibian (2015)[10]	500	-36.85 (-128.29 to 54.59)	26.71 (17.11 to 36.3)
Yap (2014)[11]	179	70 (-135.3 to 275.3)	29.95 (22.12 to 37.79)
Zerofsky (2014)[12]	57	289 (10.78 to 567.22)	30.4 (19.91 to 40.89)
Litonjua (2016)[13]	440	-14.6 (-92.94 to 63.74)	31.2 (26.46 to 35.94)
Hollis (H) (2011)[7]	335	62.8 (-145.17 to 270.77)	32.1 (20.03 to 44.17)
Valizadeh (2016)[14]	96	217 (-14.9 to 448.9)	32.7 (19.63 to 45.76)
Sablok (2015)[15]	180	200 (88.39 to 311.61)	33.89 (12.31 to 55.47)
Sahoo (H) (2016)[8]	29	0 (-350.15 to 350.15)	35.3 (20.09 to 50.51)
Dawodu (H) (2013)[6]	127	2 (-217.22 to 221.22)	41.56 (29.15 to 53.97)
Asemi (b) (2013)[16]	54	120.5 (-153.57 to 394.57)	45 (18.35 to 71.65)

Grant (L) (2014)[17]	174	28.33 (-152.21 to 208.88)	45.76 (33.84 to 57.68)
Grant (H) (2014)[17]	173	88.33 (-87.01 to 263.68)	48.26 (34.49 to 62.02)
Abotorabi (2017)[18]	110	33.7 (-139.04 to 206.44)	48.6 (39.91 to 57.29)
Hashemipour (2013)[19]	160	170.2 (42.48 to 297.92)	79.62 (71.08 to 88.16)
Roth (2013)[20]	160	14 (-138.82 to 166.82)	96 (87.4 to 104.6)
Brooke (1980)[21]	126	123 (-50.29 to 296.29)	151.8 (126.74 to 176.86)
Karamali (2015)[22]	60	172.6 (-42.78 to 387.98)	43.78 (39.6 to 47.96)
Sabet (2012)[23]	50	45 (-136.32 to 226.32)	80 (46.73 to 113.26)
Cooper (2016)[24]	1134	-37 (-104.21 to 30.21)	24.5 (21.7to27.3)

References

1. Yesiltepe Mutlu G, Ozsu E, Kalaca S, Yuksel A, Pehlevan Y, Cizmecioglu F, et al. Evaluation of Vitamin D Supplementation Doses during Pregnancy in a Population at High Risk for Deficiency. Hormone Research in Paediatrics. 2014;81(6):402-8.
2. Yu CK, Sykes L, Sethi M, Teoh TG, Robinson S. Vitamin D deficiency and supplementation during pregnancy. Clinical endocrinology. 2009;70(5):685-90. Epub 2008/09/06. doi: 10.1111/j.1365-2265.2008.03403.x.
3. Khan F, Ahmad T, Hussain R, Bhutta Z. A Randomized Controlled Trial of Oral Vitamin D Supplementation in Pregnancy to Improve Maternal Periodontal Health and Birth Weight. Journal of International Oral Health. 2016;8(6):657-65. doi: 10.2047/jioh-08-06-03.
4. Vaziri F, Nasiri S, Tavana Z, Dabbaghmanesh MH, Sharif F, Jafari P. A randomized controlled trial of vitamin D supplementation on perinatal depression: in Iranian pregnant mothers. BMC Pregnancy and Childbirth. 2016;16:239. doi: 10.1186/s12884-016-1024-7.
5. Mallet E, Gugi B, Brunelle P, Henocq A, Basuyau JP, Lemeur H. Vitamin D supplementation in pregnancy: a controlled trial of two methods. Obstet Gynecol. 1986;68(3):300-4. Epub 1986/09/01.
6. Dawodu A, Saadi HF, Bekdache G, Javed Y, Altaye M, Hollis BW. Randomized controlled trial (RCT) of vitamin D supplementation in pregnancy in a population with endemic vitamin D deficiency. The Journal of clinical endocrinology and metabolism. 2013;98(6):2337-46. Epub 2013/04/06. doi: 10.1210/jc.2013-1154.
7. Hollis BW, Johnson D, Hulsey TC, Ebeling M, Wagner CL. Vitamin D supplementation during pregnancy: double-blind, randomized clinical trial of safety and effectiveness. Journal of bone and mineral research : the official journal of the American Society for Bone and Mineral Research. 2011;26(10):2341-57. Epub 2011/06/28. doi: 10.1002/jbmr.463.
8. Sahoo SK, Katam KK, Das V, Agarwal A, Bhatia V. Maternal vitamin D supplementation in pregnancy and offspring outcomes: a double-blind randomized placebo-controlled trial. Journal of bone and mineral metabolism. 2017;35(4):464-71. Epub 2016/09/16. doi: 10.1007/s00774-016-0777-4.

9. Thiele DK, Ralph J, El-Masri M, Anderson CM. Vitamin D3 Supplementation During Pregnancy and Lactation Improves Vitamin D Status of the Mother-Infant Dyad. Journal of obstetric, gynecologic, and neonatal nursing : JOGNN. 2017;46(1):135-47. Epub 2016/11/15. doi: 10.1016/j.jogn.2016.02.016.
10. Mojibian M, Soheilykhah S, Fallah Zadeh MA, Jannati Moghadam M. The effects of vitamin D supplementation on maternal and neonatal outcome: A randomized clinical trial. Iranian Journal of Reproductive Medicine. 2015;13(11):687-96.
11. Yap C, Cheung NW, Gunton JE, Athayde N, Munns CF, Duke A, et al. Vitamin D supplementation and the effects on glucose metabolism during pregnancy: a randomized controlled trial. Diabetes care. 2014;37(7):1837-44. Epub 2014/04/25. doi: 10.2337/dc14-0155.
12. Zerofsky M, Jacoby B, Stephensen C. A randomized controlled trial of vitamin D supplementation in pregnancy: effects on vitamin D status and clinical outcomes (1041.5). The FASEB Journal. 2014;28(1_supplement):1041.5. doi: 10.1096/fasebj.28.1_supplement.1041.5.
13. Litonjua AA, Carey VJ, Laranjo N, Harshfield BJ, McElrath TF, O'Connor GT, et al. Effect of Prenatal Supplementation With Vitamin D on Asthma or Recurrent Wheezing in Offspring by Age 3 Years: The VDAART Randomized Clinical Trial. Jama. 2016;315(4):362-70. Epub 2016/01/28. doi: 10.1001/jama.2015.18589.
14. Valizadeh M, Piri Z, Mohammadian F, Kamali K, Amir Moghadami HR. The Impact of Vitamin D Supplementation on Post-Partum Glucose Tolerance and Insulin Resistance in Gestational Diabetes: A Randomized Controlled Trial. International Journal of Endocrinology and Metabolism. 2016;14(2):e34312. doi: 10.5812/ijem.34312.
15. Sablok A, Batra A, Thariani K, Batra A, Bharti R, Aggarwal AR, et al. Supplementation of vitamin D in pregnancy and its correlation with feto-maternal outcome. Clinical endocrinology. 2015;83(4):536-41. Epub 2015/02/17. doi: 10.1111/cen.12751.
16. Asemi Z, Hashemi T, Karamali M, Samimi M, Esmaillzadeh A. Effects of vitamin D supplementation on glucose metabolism, lipid concentrations, inflammation, and oxidative stress in gestational diabetes: a double-blind randomized controlled clinical trial. The American Journal of Clinical Nutrition. 2013;98(6):1425-32. doi: 10.3945/ajcn.113.072785.
17. Grant CC, Stewart AW, Scragg R, Milne T, Rowden J, Ekeroma A, et al. Vitamin D during pregnancy and infancy and infant serum 25-hydroxyvitamin D concentration. Pediatrics. 2014;133(1):e143-53. Epub 2013/12/18. doi: 10.1542/peds.2013-2602.
18. Abotorabi S, Hashemi Poor S, Esmailzadehha N, Ziaee A, Khoeiniha MH. Effect of Treatment with Vitamin D on Maternal and Neonatal Indices in Pregnant Women with Hypocalcemia: A Randomized Controlled Trial. International Journal of Pediatrics. 2017;5(9):5733-9. doi: 10.22038/ijp.2017.22146.1851.
19. Hashemipour S, Lalooha F, Zahir Mirdamadi S, Ziaee A, Dabaghi Ghaleh T. Effect of vitamin D administration in vitamin D-deficient pregnant women on maternal and neonatal serum calcium and vitamin D concentrations: a randomised clinical trial. The British journal of nutrition. 2013;110(9):1611-6. Epub 2013/05/01. doi: 10.1017/s0007114513001244.
20. Roth DE, Al Mahmud A, Raqib R, Akhtar E, Perumal N, Pezzack B, et al. Randomized placebo-controlled trial of high-dose prenatal third-trimester vitamin D3 supplementation in Bangladesh: the AViDD trial. Nutrition Journal. 2013;12:47-. doi: 10.1186/1475-2891-12-47.
21. Brooke OG, Brown IR, Bone CD, Carter ND, Cleeve HJ, Maxwell JD, et al. Vitamin D supplements in pregnant Asian women: effects on calcium status and fetal growth. British Medical Journal. 1980;280(6216):751-4.

22. Karamali M, Beihaghi E, Mohammadi AA, Asemi Z. Effects of High-Dose Vitamin D Supplementation on Metabolic Status and Pregnancy Outcomes in Pregnant Women at Risk for Pre-Eclampsia. Hormone and metabolic research = Hormon- und Stoffwechselforschung = Hormones et metabolisme. 2015;47(12):867-72. Epub 2015/05/06. doi: 10.1055/s-0035-1548835.
23. Sabet Z, Ghazi A, Tohidi M, Oladi B. VITAMIN D SUPPLEMENTATION IN PREGNANT IRANIAN WOMEN: EFFECTS ON MATERNAL AND NEONATAL VITAMIN D AND PARATHYROID HORMONE STATUS. Acta Endocrinologica (1841-0987). 2012;8(1).
24. Cooper C, Harvey NC, Bishop NJ, Kennedy S, Papageorghiou AT, Schoenmakers I, et al. Maternal gestational vitamin D supplementation and offspring bone health (MAVIDOS): a multicentre, double-blind, randomised placebo-controlled trial. The Lancet Diabetes & Endocrinology. 2016;4(5):393-402. doi: 10.1016/S2213-8587(16)00044-9.
