# Supplementary material for: Association of maternal circulating 25(OH)D and calcium with birth weight: A mendelian randomisation analysis
Source: PLoS Med. 2019 Jun 18;16(6):e1002828. doi: 10.1371/journal.pmed.1002828 (PMC6581250; doi:10.1371/journal.pmed.1002828)
Supplement: S4 Table — RCT, randomised controlled trial. (PDF) [file pmed.1002828.s010.pdf]

S4 Table: Studies used to calculate the RCT instrumental variable effect of calcium on birth weight

Studies	Number of participants in the study	Mean Difference in birth weight between supplementation and placebo group, in g	Mean Difference in calcium levels between supplementation and placebo group, in mg/dl
Boggess (1997)[1]	18	82 (-97.3 to 261.3)	0.1 (-6.262 to 6.462)
Chan (2006)[2]	41	15 (-89.87 to 119.87)	0.1 (-0.51 to 0.71)
Lopez-Jaramillo (1989)[3]	92	265 (142.5 to 287.5)	0.16 (-0.12 to 0.44)
Lopez-Jaramillo (1997)[4]	260	110 (73.44 to 146.56)	0.28 (0.28 to 0.28)
Belizan (1983) (1g supplement)[5]	16	-354.000 (-751.35 to 43.35)	0.42 (-1.97 to 2.81)
Belizan (1983) (2g supplement)[5]	16	42.000 (-268.82 to 352.82)	0.94 (-2.14 to 4.02)
Wanchu 2001[6]	100	100.000 (-77.48 to 277.48)	0.3 (-0.39 to 0.99)

The RCTs used in the analyses of the effect of gestational circulating 25(OH)D on birth weight were identified from the systematic review by Buppasiri et al 2015[7]

References

1. Boggess KA, Samuel L, Schmucker BC, Waters J, Easterling TR. A randomized controlled trial of the effect of third-trimester calcium supplementation on maternal hemodynamic function. Obstet Gynecol. 1997;90(2):157-61. Epub 1997/08/01. doi: 10.1016/s0029-7844(97)00248-2.
2. Chan GM, McElligott K, McNaught T, Gill G. Effects of dietary calcium intervention on adolescent mothers and newborns: A randomized controlled trial. Obstet Gynecol. 2006;108(3 Pt 1):565-71. Epub 2006/09/02. doi: 10.1097/01.AOG.0000231721.42823.9e.
3. P. LJ, M. N, M. WR, R. Y. Calcium supplementation reduces the risk of pregnancy-induced hypertension in an Andes population. BJOG: An International Journal of Obstetrics & Gynaecology. 1989;96(6):648-55. doi: doi:10.1111/j.1471-0528.1989.tb03278.x.
4. Lopez-Jaramillo P, Delgado F, Jacome P, Teran E, Ruano C, Rivera J. Calcium supplementation and the risk of preeclampsia in Ecuadorian pregnant teenagers. Obstet Gynecol. 1997;90(2):162-7. Epub 1997/08/01. doi: 10.1016/s0029-7844(97)00254-8.

5. Belizan JM, Villar J, Zalazar A, Rojas L, Chan D, Bryce GF. Preliminary evidence of the effect of calcium supplementation on blood pressure in normal pregnant women. American journal of obstetrics and gynecology. 1983;146(2):175-80. Epub 1983/05/15.

6. Wanchu M, Malhotra S, Khullar M. Calcium supplementation in pre-eclampsia. The Journal of the Association of Physicians of India. 2001;49:795-8. Epub 2002/02/12.

7. Buppasiri P, Lumbiganon P, Thinkhamrop J, Ngamjarus C, Laopaiboon M, Medley N. Calcium supplementation (other than for preventing or treating hypertension) for improving pregnancy and infant outcomes. Cochrane Database of Systematic Reviews. 2015;(2). doi: 10.1002/14651858.CD007079.pub3.
