# Supplementary material for: Association of maternal circulating 25(OH)D and calcium with birth weight: A mendelian randomisation analysis
Source: PLoS Med. 2019 Jun 18;16(6):e1002828. doi: 10.1371/journal.pmed.1002828 (PMC6581250; doi:10.1371/journal.pmed.1002828)
Supplement: S5 Table — SNP, single-nucleotide polymorphism. (PDF) [file pmed.1002828.s011.pdf]

S5 Table:SNP effects on first child birth weight in all studies

SNP	Study	SNP-outcome effect (g)
rs10741657	UK Biobank (N=190,406)	1.517 (-1.567 to 4.601)
	ALSPAC (N=4,576)	12.076 (-6.313 to 30.464)
	EFSOCH (N=647)	-7.120 (-54.209 to 39.968)
rs117913124	UK Biobank (N=190,406)	-1.121(-10.34 to 8.097)
	ALSPAC (N=4,576)	0.244 (-55.124 to 55.613)
	EFSOCH (N=647)	19.734 (-123.241 to 162.71)
rs12785878	UK Biobank (N=190,406)	2.030 (-1.682 to 5.742)
	ALSPAC (N=4,576)	15.855 (-5.217 to 36.927)
	EFSOCH (N=647)	-0.542 (-57.256 to 56.173)
rs3755967	UK Biobank (N=190,406)	-0.756 (-4.09 to 2.578)
	ALSPAC (N=4,576)	9.447 (-10.41 to 29.303)
	EFSOCH (N=647)	34.727 (-17.749 to 87.202)
rs17216707	UK Biobank (N=190,406)	1.943 (-2.001 to 5.888)
	ALSPAC (N=4,576)	0.926 (-22.32 to 24.173)
	EFSOCH (N=647)	2.150 (-63.878 to 68.177)
rs10745742	UK Biobank (N=190,406)	-1.813 (-4.951 to 1.324)
	ALSPAC (N=4,576)	-1.352 (-20.246 to 17.543)
	EFSOCH (N=647)	-34.556 (-82.902 to 13.789)
rs8018720	UK Biobank (N=190,406)	-1.862 (-5.835 to 2.111)
	ALSPAC (N=4,576)	6.977 (-16.75 to 30.704)
	EFSOCH (N=647)	13.136 (-50.613 to 76.885)
rs1801725	UK Biobank (N=190,406)	1.349 (-3.166 to 5.865)
	ALSPAC (N=4,576)	31.759 (4.884 to 58.634)
	EFSOCH (N=647)	-18.180 (-87.642 to 51.282)
rs1550532	UK Biobank (N=190,406)	-2.579 (-5.832 to 0.674)
	ALSPAC (N=4,576)	-6.095 (-25.7 to 13.511)
	EFSOCH (N=647)	-1.471 (-53.177 to 50.236)
rs780094	UK Biobank (N=190,406)	-5.379 (-8.495 to -2.263)

	ALSPAC (N=4,576)	-1.747 (-20.415 to 16.921)
	EFSOCH (N=647)	-24.770 (-70.675 to 21.135)
rs10491003	UK Biobank (N=190,406)	1.169 (-4.065 to 6.403)
	ALSPAC (N=4,576)	-0.579 (-32.680 to 31.521)
	EFSOCH (N=647)	-19.229 (-100.591 to 62.133)
rs7481584	UK Biobank (N=190,406)	-2.195 (-5.553 to 1.164)
	ALSPAC (N=4,576)	-4.468 (-24.868 to 15.932)
	EFSOCH (N=647)	-1.612 (-56.211 to 52.987)
rs7336933	UK Biobank (N=190,406)	-3.252 (-7.474 to 0.97)
	ALSPAC (N=4,576)	6.193 (-19.201 to 31.586)
	EFSOCH (N=647)	-42.09 (-108.322 to 24.142)
rs1570669	UK Biobank (N=190,406)	-0.573 (-3.781 to 2.636)
	ALSPAC (N=4,576)	-9.767 (-28.881 to 9.347)
	EFSOCH (N=647)	21.217 (-28.934 to 71.367)
