# Supplementary material for: Association of maternal circulating 25(OH)D and calcium with birth weight: A mendelian randomisation analysis
Source: PLoS Med. 2019 Jun 18;16(6):e1002828. doi: 10.1371/journal.pmed.1002828 (PMC6581250; doi:10.1371/journal.pmed.1002828)
Supplement: S6 Table — SNP, single-nucleotide polymorphism. (PDF) [file pmed.1002828.s012.pdf]

S6 Table: SNP effects on own birth weight in UK Biobank (N=215,444)

SNP	SNP-outcome effect (g)
rs10741657	4.083 (1.178 to 6.987)
rs117913124	-3.97 (-12.703 to 4.763)
rs12785878	3.349 (-0.134 to 6.831)
rs3755967	0.652 (-2.487 to 3.792)
rs17216707	4.041 (0.339 to 7.742)
rs10745742	-0.533 (-3.479 to 2.412)
rs8018720	-1.492 (-5.232 to 2.249)
rs1801725	-6.466 (-10.715 to -2.217)
rs1550532	-5.091 (-8.151 to -2.03)
rs780094	-1.948 (-4.878 to 0.983)
rs10491003	2.010 (-2.932 to 6.951)
rs7481584	1.236 (-1.923 to 4.396)
rs7336933	0.208 (-3.769 to 4.186)
rs1570669	3.407 (0.392 to 6.423)
