# Supplementary material for: Association of maternal circulating 25(OH)D and calcium with birth weight: A mendelian randomisation analysis
Source: PLoS Med. 2019 Jun 18;16(6):e1002828. doi: 10.1371/journal.pmed.1002828 (PMC6581250; doi:10.1371/journal.pmed.1002828)
Supplement: S7 Table — ALSPAC, Avon Longitudinal Study of Parents and Children; EFSOCH, Exeter Family Study of Childhood Health; SNP, single-nucleotide polymorphism. (PDF) [file pmed.1002828.s013.pdf]

S7 Table: SNP effects on fetal adjusted birth weight in ALSPAC and EFSOCH

SNP	Study	SNP-outcome effect (g)
rs10741657	ALSPAC (N=4,576)	12.325 (-8.705 to 33.355)
	EFSOCH (N=647)	-21.514 (-76.659 to 33.631)
rs117913124	ALSPAC (N=4,576)	9.165 (-56.077 to 74.407)
	EFSOCH (N=647)	11.226 (-147.894 to 170.345)
rs12785878	ALSPAC (N=4,576)	27.337 (3.041 to 51.633)
	EFSOCH (N=647)	-15.656 (-80.016 to 48.704)
rs3755967	ALSPAC (N=4,576)	13.299 (-9.578 to 36.176)
	EFSOCH (N=647)	42.838 (-18.736 to 104.412)
rs17216707	ALSPAC (N=4,576)	-0.508 (-27.622 to 26.607)
	EFSOCH (N=647)	-12.660 (-87.102 to 61.782)
rs10745742	ALSPAC (N=4,576)	-3.128 (-24.790 to 18.533)
	EFSOCH (N=647)	-59.398 (-116.456 to -2.34)
rs8018720	ALSPAC (N=4,576)	-3.236 (-30.492 to 24.019)
	EFSOCH (N=647)	20.294 (-53.447 to 94.034)
rs1801725	ALSPAC (N=4,576)	31.476 (0.706 to 62.246)
	EFSOCH (N=647)	-43.012 (-120.582 to 34.558)
rs1550532	ALSPAC (N=4,576)	5.037 (-17.553 to 27.627)
	EFSOCH (N=647)	-16.937 (-77.309 to 43.435)
rs780094	ALSPAC (N=4,576)	8.070 (-13.375 to 29.515)
	EFSOCH (N=647)	-31.69 (-85.520 to 22.139)
rs10491003	ALSPAC (N=4,576)	-6.140 (-42.415 to 30.134)
	EFSOCH (N=647)	-48.253 (-139.743 to 43.236)
rs7481584	ALSPAC (N=4,576)	-2.332 (-25.799 to 21.135)
	EFSOCH (N=647)	-4.990 (-67.296 to 57.317)
rs7336933	ALSPAC (N=4,576)	2.363 (-26.832 to 31.558)
	EFSOCH (N=647)	-17.501 (-94.267 to 59.265)
rs1570669	ALSPAC (N=4,576)	-17.949 (-39.990 to 4.092)
	EFSOCH (N=647)	10.717 (-47.005 to 68.439)
