# Supplementary material for: Association of maternal circulating 25(OH)D and calcium with birth weight: A mendelian randomisation analysis
Source: PLoS Med. 2019 Jun 18;16(6):e1002828. doi: 10.1371/journal.pmed.1002828 (PMC6581250; doi:10.1371/journal.pmed.1002828)
Supplement: S8 Table — IV, instrumental variable; RCT, randomised controlled trial. (PDF) [file pmed.1002828.s014.pdf]

S8 Table: Risk of Bias in studies included in IV of RCT analyses of Vitamin D supplementation

Study	Number randomised	Loss to follow-up (%)[a]	Placebo control	Intention to treat analysis[a]	Risk of Bias[b]						
					RSG	AC	BP	BO	IOD	SR	Other
Abotorabi (2017)[1]	110	23	No	Unclear	Low	Unclear	High	Unclear	Low	High	Low
Asemi (2013)[2]	54	11	Yes	Yes	Low	Low	Low	Low	Low	Low	Low
Brooke (1980)[3]	130 analysed	Unclear	Yes	Unclear	Unclear	Unclear	Low	Low	Low	Low	Low
Cooper (2016)[4]	1134	15	No	Low	Low	Low	Low	Low	Low	Low	Low
Dawodu (L) (2013)[5]	129	17	No	No	Low	Low	Low	Low	High	High	Low
Dawodu (H) (2013)[5]	127	13	No	No	Low	Low	Low	Low	High	High	Low
Grant (L) (2014)[6]	174	3	Yes	Unclear	Low	Low	Low	Low	Low	Low	Low
Grant (H) (2014)[6]	173	3	Yes	Unclear	Low	Low	Low	Low	Low	Low	Low
Hashemipour (2013)[7]	130	6	No	No	Low	Low	High	High	Low	Low	Low
Hollis (L) (2011)[8]	333	30	Yes	No	High	Low	Low	Low	Low	Low	Low
Hollis (H) (2011)[8]	335	32	Yes	No	High	Low	Low	Low	Low	Low	Low
Karamali (2015)[9]	30 analysed	Unclear	Yes	Unclear	Low	Low	Low	Low	Low	Low	Low

Khan (2016)[10]	85	Unclear	Yes	Unclear	Unclear	Low	Low	Low	Unclear	Low	Low
Litonjua (2016)[11]	881	8	No	No	Low	Unclear	Low	Low	Low	Low	Low
Mallet (L) (1986)[12][c]	Unclear (36 analysed)	Unclear	Yes	Unclear	Low	Unclear	High	Low	Low	Low	Low
Mallet (H) (1986)[12][c]	Unclear (30 analysed)	Unclear	Yes	Unclear	Low	Unclear	High	Low	Low	Low	Low
Mojibian (2015)[13]	500	22	No	No	Low	Low	High	High	High	Low	Low
Mutlu (L) 2014[14]	59	24	No	No	Unclear	Unclear	High	High	Low	Low	Low
Mutlu (H) (2014)[14]	60	27	No	No	Unclear	Unclear	High	High	Low	Low	Low
Roth (2013)[15]	160	8	Yes	No	Low	Low	Low	Low	Low	Low	Low
Sabet (2012)[16]	50 analysed	Unclear	Yes	Unclear	Unclear	Unclear	Low	Low	Low	Low	Low
Sablok (2015)[17]	180	8	No	No	Low	Unclear	High	Unclear	Unclear	High	Low
Sahoo (L) (2016)[18]	200	85[d]	Yes	No	Low	Low	Low	Low	High	Low	Low
Sahoo (H) (2016)[18]	200	80[d]	No	No	Low	Low	Low	Low	High	Low	Low
Thiele (2016)[19]	16	19	Yes	Yes	Unclear	Unclear	Low	Low	Unclear	Unclear	Low
Yap (2014)[20]	169	6	No	No	High	High	Low	Low	Low	High	Low
Valizadeh (2016)[21]	96	6	No	Unclear	Unclear	Low	High	High	Low	Low	Low
Vaziri (2016)[22]	153	17%	No	No	Unclear	Unclear	Low	Low	Low	High	Low

Yu (L) (2009)[23]	120	1	No	Unclear	Low	High	High	High	Low	Low	Low
Yu (H) 2009[23]	120	1	No	Unclear	Low	High	High	High	Low	Low	Low
Zerofsky (2014)[24]	57	14	Yes	No	Low	Low	Low	Low	Low	Low	Low

Note there are 30 rows in this table which reflects the 30 instrumental variable estimates from the 24 RCTs that were included in these analyses; two IV estimates were obtained from six trials in which participants were randomised to one of three groups: low dose vitamin D (L), high dose vitamin D (H) or control

[a] All entries relate to analyses of birth weight (birth weight is often not the primary outcome in these trials and the results may be different for the primary outcomes)

[b] Each of the seven categories were categorised by reviewers as low, medium, or high risk of bias or unclear. RSG: Random Sequence Generation; AC: Allocation Concealment; BP: Blinding of personnel/participants (performance bias); BO: Blinding of outcome assessment (detection bias); IOD: Incomplete outcome data (attrition bias); SR: Selective Reporting; Other: other sources of bias.

[c] For this study we were only able to access the abstracts and not the full papers

[d] This study was primarily concerned with outcomes at 12-16 months and over 80% were lost to follow-up by this age (47% had been lost to follow-up at birth but differences in BW between randomised groups was only presented in those included in the 12-16 months follow-up).

References

1. Abotorabi S, Hashemi Poor S, Esmailzadehha N, Ziaee A, Khoeiniha MH. Effect of Treatment with Vitamin D on Maternal and Neonatal Indices in Pregnant Women with Hypocalcemia: A Randomized Controlled Trial. International Journal of Pediatrics. 2017;5(9):5733-9. doi: 10.22038/ijp.2017.22146.1851.
2. Asemi Z, Hashemi T, Karamali M, Samimi M, Esmaillzadeh A. Effects of vitamin D supplementation on glucose metabolism, lipid concentrations, inflammation, and oxidative stress in gestational diabetes: a double-blind randomized controlled clinical trial. The American Journal of Clinical Nutrition. 2013;98(6):1425-32. doi: 10.3945/ajcn.113.072785.
3. Brooke OG, Brown IR, Bone CD, Carter ND, Cleeve HJ, Maxwell JD, et al. Vitamin D supplements in pregnant Asian women: effects on calcium status and fetal growth. British Medical Journal. 1980;280(6216):751-4.

4. Cooper C, Harvey NC, Bishop NJ, Kennedy S, Papageorghiou AT, Schoenmakers I, et al. Maternal gestational vitamin D supplementation and offspring bone health (MAVIDOS): a multicentre, double-blind, randomised placebo-controlled trial. The Lancet Diabetes & Endocrinology. 2016;4(5):393-402. doi: 10.1016/S2213-8587(16)00044-9.
5. Dawodu A, Saadi HF, Bekdache G, Javed Y, Altaye M, Hollis BW. Randomized controlled trial (RCT) of vitamin D supplementation in pregnancy in a population with endemic vitamin D deficiency. The Journal of clinical endocrinology and metabolism. 2013;98(6):2337-46. Epub 2013/04/06. doi: 10.1210/jc.2013-1154.
6. Grant CC, Stewart AW, Scragg R, Milne T, Rowden J, Ekeroma A, et al. Vitamin D during pregnancy and infancy and infant serum 25-hydroxyvitamin D concentration. Pediatrics. 2014;133(1):e143-53. Epub 2013/12/18. doi: 10.1542/peds.2013-2602.
7. Hashemipour S, Lalooha F, Zahir Mirdamadi S, Ziaee A, Dabaghi Ghaleh T. Effect of vitamin D administration in vitamin D-deficient pregnant women on maternal and neonatal serum calcium and vitamin D concentrations: a randomised clinical trial. The British journal of nutrition. 2013;110(9):1611-6. Epub 2013/05/01. doi: 10.1017/s0007114513001244.
8. Hollis BW, Johnson D, Hulsey TC, Ebeling M, Wagner CL. Vitamin D supplementation during pregnancy: double-blind, randomized clinical trial of safety and effectiveness. Journal of bone and mineral research : the official journal of the American Society for Bone and Mineral Research. 2011;26(10):2341-57. Epub 2011/06/28. doi: 10.1002/jbmr.463.
9. Karamali M, Beihaghi E, Mohammadi AA, Asemi Z. Effects of High-Dose Vitamin D Supplementation on Metabolic Status and Pregnancy Outcomes in Pregnant Women at Risk for Pre-Eclampsia. Hormone and metabolic research = Hormon- und Stoffwechselforschung = Hormones et metabolisme. 2015;47(12):867-72. Epub 2015/05/06. doi: 10.1055/s-0035-1548835.
10. Khan F, Ahmad T, Hussain R, Bhutta Z. A Randomized Controlled Trial of Oral Vitamin D Supplementation in Pregnancy to Improve Maternal Periodontal Health and Birth Weight. Journal of International Oral Health. 2016;8(6):657-65. doi: 10.2047/jioh-08-06-03.
11. Litonjua AA, Carey VJ, Laranjo N, Harshfield BJ, McElrath TF, O'Connor GT, et al. Effect of Prenatal Supplementation With Vitamin D on Asthma or Recurrent Wheezing in Offspring by Age 3 Years: The VDAART Randomized Clinical Trial. Jama. 2016;315(4):362-70. Epub 2016/01/28. doi: 10.1001/jama.2015.18589.
12. Mallet E, Gugi B, Brunelle P, Henocq A, Basuyau JP, Lemeur H. Vitamin D supplementation in pregnancy: a controlled trial of two methods. Obstet Gynecol. 1986;68(3):300-4. Epub 1986/09/01.
13. Mojibian M, Soheilykhah S, Fallah Zadeh MA, Jannati Moghadam M. The effects of vitamin D supplementation on maternal and neonatal outcome: A randomized clinical trial. Iranian Journal of Reproductive Medicine. 2015;13(11):687-96.
14. Yesiltepe Mutlu G, Ozsu E, Kalaca S, Yuksel A, Pehlevan Y, Cizmecioglu F, et al. Evaluation of Vitamin D Supplementation Doses during Pregnancy in a Population at High Risk for Deficiency. Hormone Research in Paediatrics. 2014;81(6):402-8.
15. Roth DE, Al Mahmud A, Raqib R, Akhtar E, Perumal N, Pezzack B, et al. Randomized placebo-controlled trial of high-dose prenatal third-trimester vitamin D3 supplementation in Bangladesh: the AViDD trial. Nutrition Journal. 2013;12:47-. doi: 10.1186/1475-2891-12-47.
16. Sabet Z, Ghazi A, Tohidi M, Oladi B. VITAMIN D SUPPLEMENTATION IN PREGNANT IRANIAN WOMEN: EFFECTS ON MATERNAL AND NEONATAL VITAMIN D AND PARATHYROID HORMONE STATUS. Acta Endocrinologica (1841-0987). 2012;8(1).

17. Sablok A, Batra A, Thariani K, Batra A, Bharti R, Aggarwal AR, et al. Supplementation of vitamin D in pregnancy and its correlation with feto-maternal outcome. Clinical endocrinology. 2015;83(4):536-41. Epub 2015/02/17. doi: 10.1111/cen.12751.
18. Sahoo SK, Katam KK, Das V, Agarwal A, Bhatia V. Maternal vitamin D supplementation in pregnancy and offspring outcomes: a double-blind randomized placebo-controlled trial. Journal of bone and mineral metabolism. 2017;35(4):464-71. Epub 2016/09/16. doi: 10.1007/s00774-016-0777-4.
19. Thiele DK, Ralph J, El-Masri M, Anderson CM. Vitamin D3 Supplementation During Pregnancy and Lactation Improves Vitamin D Status of the Mother-Infant Dyad. Journal of obstetric, gynecologic, and neonatal nursing : JOGNN. 2017;46(1):135-47. Epub 2016/11/15. doi: 10.1016/j.jogn.2016.02.016.
20. Yap C, Cheung NW, Gunton JE, Athayde N, Munns CF, Duke A, et al. Vitamin D supplementation and the effects on glucose metabolism during pregnancy: a randomized controlled trial. Diabetes care. 2014;37(7):1837-44. Epub 2014/04/25. doi: 10.2337/dc14-0155.
21. Valizadeh M, Piri Z, Mohammadian F, Kamali K, Amir Moghadami HR. The Impact of Vitamin D Supplementation on Post-Partum Glucose Tolerance and Insulin Resistance in Gestational Diabetes: A Randomized Controlled Trial. International Journal of Endocrinology and Metabolism. 2016;14(2):e34312. doi: 10.5812/ijem.34312.
22. Vaziri F, Nasiri S, Tavana Z, Dabbaghmanesh MH, Sharif F, Jafari P. A randomized controlled trial of vitamin D supplementation on perinatal depression: in Iranian pregnant mothers. BMC Pregnancy and Childbirth. 2016;16:239. doi: 10.1186/s12884-016-1024-7.
23. Yu CK, Sykes L, Sethi M, Teoh TG, Robinson S. Vitamin D deficiency and supplementation during pregnancy. Clinical endocrinology. 2009;70(5):685-90. Epub 2008/09/06. doi: 10.1111/j.1365-2265.2008.03403.x.
24. Zerofsky M, Jacoby B, Stephensen C. A randomized controlled trial of vitamin D supplementation in pregnancy: effects on vitamin D status and clinical outcomes (1041.5). The FASEB Journal. 2014;28(1_supplement):1041.5. doi: 10.1096/fasebj.28.1_supplement.1041.5.
