# Supplementary material for: Association of maternal circulating 25(OH)D and calcium with birth weight: A mendelian randomisation analysis
Source: PLoS Med. 2019 Jun 18;16(6):e1002828. doi: 10.1371/journal.pmed.1002828 (PMC6581250; doi:10.1371/journal.pmed.1002828)
Supplement: S9 Table — IV, instrumental variable; RCT, randomised controlled trial. (PDF) [file pmed.1002828.s015.pdf]

S9 Table: Risk of Bias in studies included in IV of RCT analyses of Calcium supplementation

Study	Number randomised	Loss to follow-up (%)	Placebo control	Intention to treat analysis	Risk of Bias*						
					RSG	AC	BP	BO	IOD	SR	Other
Bogges 1997[1]	23	22%	Yes	No	low	low	low	low	unclear	low	low
Belizan 1983[2]	36	Unclear '0 missing data'	Yes	Yes	unclear	unclear	low	low	low	low	low
Chan 2006[3]**	72	8%	No	No	low	low	high	high	low	unclear	low
Lopez-Jaramillo 1989[4]	106	13%	Yes	Yes	low	low	low	low	unclear	unclear	low
Lopez-Jaramillo 1997[5]	274	5%	Yes	No	low	low	low	low	low	low	low
Wanchu 2001[6]	120	17%	No	No	unclear	unclear	high	high	high	unclear	low

* Each of the seven categories were categorised by reviewers as low, medium, or high risk of bias or unclear. RSG: Random Sequence Generation; AC: Allocation Concealment; BP: Blinding of personnel/participants (performance bias); BO: Blinding of outcome assessment (detection bias); IOD: Incomplete outcome data (attrition bias); SR: Selective Reporting; Other: other sources of bias

** Chan participants were randomised to control or one of two intervention groups: orange juice with calcium fortification or increased diary intake (only the comparison of orange just with calcium fortification to control group was used here); the other three studies compared calcium supplementation tablets to placebo or no supplementation (control groups)

References

1. Boggess KA, Samuel L, Schmucker BC, Waters J, Easterling TR. A randomized controlled trial of the effect of third-trimester calcium supplementation on maternal hemodynamic function. Obstetrics & Gynecology. 1997;90(2):157-61. doi: https://doi.org/10.1016/S0029-7844(97)00248-2.
2. Belizan JM, Villar J, Zalazar A, Rojas L, Chan D, Bryce GF. Preliminary evidence of the effect of calcium supplementation on blood pressure in normal pregnant women. American journal of obstetrics and gynecology. 1983;146(2):175-80. Epub 1983/05/15.
3. Chan GM, McElligott K, McNaught T, Gill G. Effects of dietary calcium intervention on adolescent mothers and newborns: A randomized controlled trial. Obstet Gynecol. 2006;108(3 Pt 1):565-71. Epub 2006/09/02. doi: 10.1097/01.AOG.0000231721.42823.9e.
4. P. LJ, M. N, M. WR, R. Y. Calcium supplementation reduces the risk of pregnancy-induced hypertension in an Andes population. BJOG: An International Journal of Obstetrics & Gynaecology. 1989;96(6):648-55. doi: doi:10.1111/j.1471-0528.1989.tb03278.x.
5. Lopez-Jaramillo P, Delgado F, Jacome P, Teran E, Ruano C, Rivera J. Calcium supplementation and the risk of preeclampsia in Ecuadorian pregnant teenagers. Obstet Gynecol. 1997;90(2):162-7. Epub 1997/08/01. doi: 10.1016/s0029-7844(97)00254-8.
6. Wanchu M, Malhotra S, Khullar M. Calcium supplementation in pre-eclampsia. The Journal of the Association of Physicians of India. 2001;49:795-8. Epub 2002/02/12.
