# Supplementary material for: Association of maternal circulating 25(OH)D and calcium with birth weight: A mendelian randomisation analysis
Source: PLoS Med. 2019 Jun 18;16(6):e1002828. doi: 10.1371/journal.pmed.1002828 (PMC6581250; doi:10.1371/journal.pmed.1002828)
Supplement: S10 Table — (PDF) [file pmed.1002828.s016.pdf]

S10 Table: Associations between weighted-allele-scores and potential confounders

	Study	Difference in mean or odds ratio (95% CI) per unit increase in weighted allele score (WAS)	
		25(OH)D	Calcium
Maternal BMI (kg/m^2)*	UK Biobank (N = 243,797)[c,e]	0.001 (-0.007 to 0.009)	0.001 (-0.002 to 0.005)
	ALSPAC (N =6,544)	-0.020 (-0.073 to 0.034)	-0.008 (-0.030 to 0.014)
	EFSOCH (N =844)	0.102 (-0.042 to 0.247)	0.031 (-0.030 to 0.092)
Maternal Height (m)*	UK Biobank (N = 244,363)[d,e]	-0.010 (-0.017 to -0.003)	-0.002 (-0.005 to 0.001)
	ALSPAC (N =6,862)	0.020 (-0.032 to 0.073)	-0.004 (-0.026 to 0.018)
	EFSOCH (N =930)	0.163 (0.024 to 0.302)	0.026 (-0.032 to 0.084)
Maternal Systolic Blood Pressure (mmHg)*	UK Biobank (N = 244,183)[e]	-0.002 (-0.009 to 0.006)	-0.001 (-0.004 to 0.002)
	ALSPAC (N =1,451)	-0.033 (-0.150 to 0.084)	-0.033 (-0.080 to 0.014)
	EFSOCH (N =NA)	NA	NA
Maternal Townsend area deprivation*[a]	UK Biobank (N = 244,564)	0.002 (-0.006 to 0.010)	0.004 (0.000 to 0.007)
	ALSPAC (N =NA)	NA	NA
	EFSOCH (N =933)	-0.196 (-0.336 to -0.055)	-0.016 (-0.075 to 0.043)
Education (odds ratio of university degree yes or no)	UK Biobank (N = 238,261)[e]	0.997 (0.979 to 1.014)	0.987 (0.980 to 0.995)
	ALSPAC (N =6,956)	1.032 (0.876 to 1.188)	0.955 (0.897 to 1.012)
	EFSOCH (N =NA)	NA	NA
Maternal smoking (odds ratio of current smoker vs non-smoker)	UK Biobank (N = 160,043)[e]	0.983 (0.951 to 1.015)	1.008 (0.994 to 1.022)
	ALSPAC (N =7,237)	0.990 (0.859 to 1.122)	1.007 (0.950 to 1.065)
	EFSOCH (N =928)	1.233 (0.549 to 1.917)	1.001 (0.831 to 1.172)
Western Diet[b]	UK Biobank (N = 355,829)[e]	-0.001 (-0.008 to 0.006)	0.001 (-0.002 to 0.004)
	ALSPAC (N =NA)	NA	NA
	EFSOCH (N =NA)	NA	NA

*The values given are z-scores/single inverse normalized. a) A score that takes values from the census of an area (namely percentage of households without a motor vehicle, percentage of households with more than one person per room, percentage of households not owner-occupied and percentage of residents who are unemployed), converts them to z-scores, then adds the values up, with a greater value meaning

greater deprivation. b) Western Diet is a principal component of variation in reported diet in UK Biobank. c) The UK Biobank female BMI variable excluded those with a BMI <15 and those that were pregnant, the variable then being adjusted for age, assessment centre and five principal components, with the residuals being extracted and inverse-normalized. d) The UK Biobank female Height variable was adjusted for age, assessment centre and five principal components, with the residuals being extracted and inverse-normalized. e) All UK Biobank variables were adjusted for five principal components
