# Supplementary material for: Association of maternal circulating 25(OH)D and calcium with birth weight: A mendelian randomisation analysis
Source: PLoS Med. 2019 Jun 18;16(6):e1002828. doi: 10.1371/journal.pmed.1002828 (PMC6581250; doi:10.1371/journal.pmed.1002828)
Supplement: S11 Table — MR, mendelian randomisation. (PDF) [file pmed.1002828.s017.pdf]

S11 Table: Multivariable MR for 25(OH)D and calcium effect on birth weight in UK Biobank (adjusting for height effects)

MR Model	Exposure-outcome effect (g)
IVW for 25(OH)D (main result)	-0.03 (-3.08 to 3.03)
IVW leaving rs117913124 out[a]	0.22 (-3.74 to 4.17)
Multivariable model IVW for 25(OH)D with exposure and height SNPs adjusted for height [b]	-0.60 (-4.79 to 1.95)
IVW for calcium (main result)	-20 (-50 to 11)
Partial multivariable model for calcium [c]	-45 (-97 to 7)

[a] There was no summary data for rs117913124 (one of the 25(OH)D genetic instruments), or any proxies, in the GWAS of height used for this multivariable MR analysis[1] therefore we have included in this table both the main unadjusted IVW result and also the result with rs117913124 left out so that we can compare the multivariable IVW adjusted for height to both the main results and one with rs117913124 left out (as this has to be left out in the multivariable MR analyses) [b] Multivariable IVW MR analyses in which we adjust the 25(OH)D-BW effect for the potential confounding effect of height. [c] In these analyses we were only able to adjust for maternal education by including the summary difference in mean education for each calcium (genetic instrument) SNP along with their difference in mean calcium.

References

1. Wood AR, Esko T, Yang J, Vedantam S, Pers TH, Gustafsson S, et al. Defining the role of common variation in the genomic and biological architecture of adult human height. Nature genetics. 2014;46:1173. doi: 10.1038/ng.3097.
