# Supplementary material for: Association of maternal circulating 25(OH)D and calcium with birth weight: A mendelian randomisation analysis
Source: PLoS Med. 2019 Jun 18;16(6):e1002828. doi: 10.1371/journal.pmed.1002828 (PMC6581250; doi:10.1371/journal.pmed.1002828)
Supplement: S1 Fig — ALSPAC, Avon Longitudinal Study of Parents and Children; EFSOCH, Exeter Family Study of Childhood Health. (PDF) [file pmed.1002828.s018.pdf]

S1 Fig: Flow diagram of participant inclusion for ALSPAC and EFSOCH

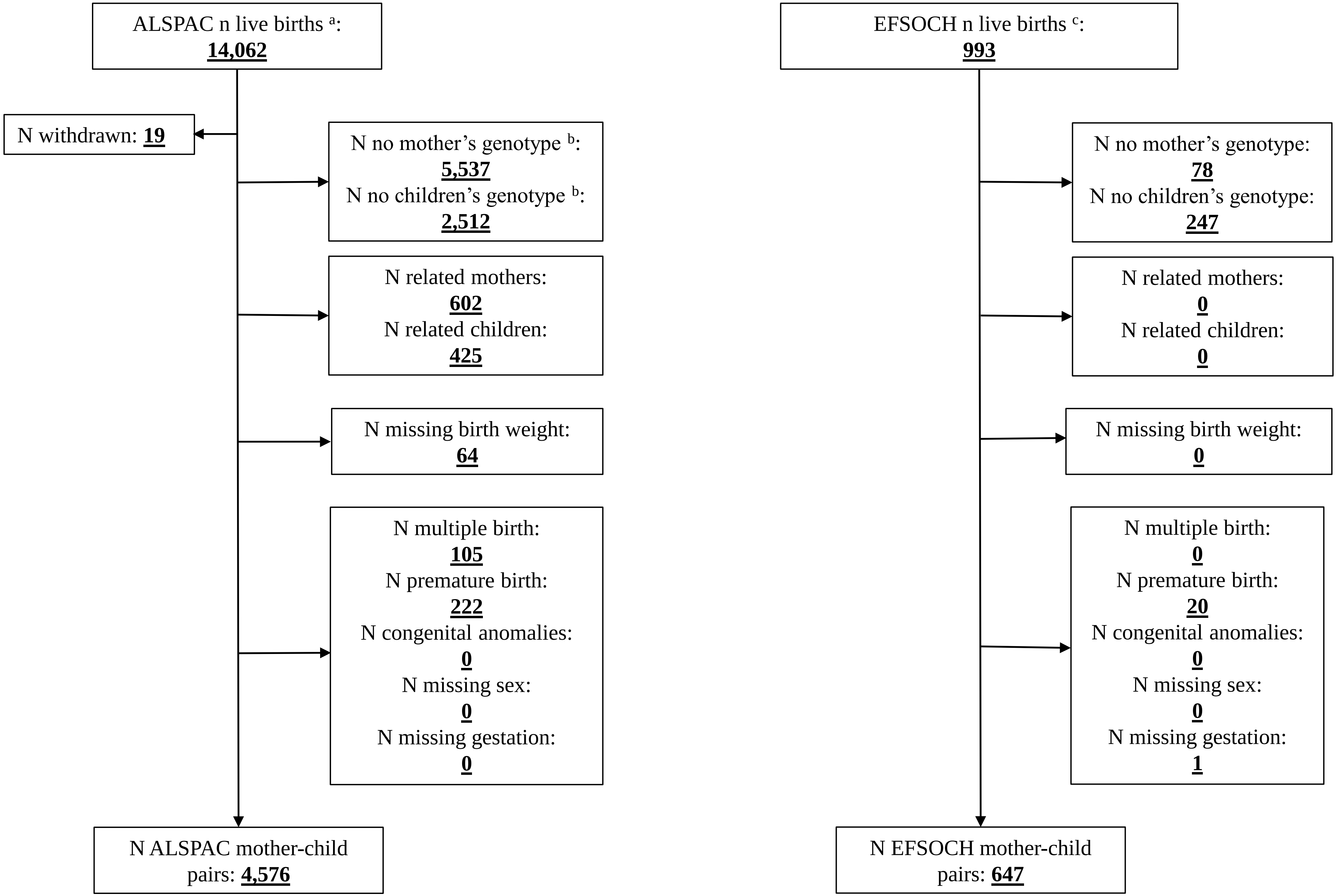

a) All participants were recruited as part of the core phase and were born alive.

b) All genotyped participants were White European.

c) All participants were born alive; they were also all White European (recruited at onset) and related individuals and twins were pre-excluded.
