# Supplementary figures and images for: Association of maternal circulating 25(OH)D and calcium with birth weight: A mendelian randomisation analysis
Source: PLoS Med. 2019 Jun 18;16(6):e1002828. doi: 10.1371/journal.pmed.1002828 (PMC6581250; doi:10.1371/journal.pmed.1002828)

**S2 Fig: Flow diagram for participant inclusion in UK Biobank**

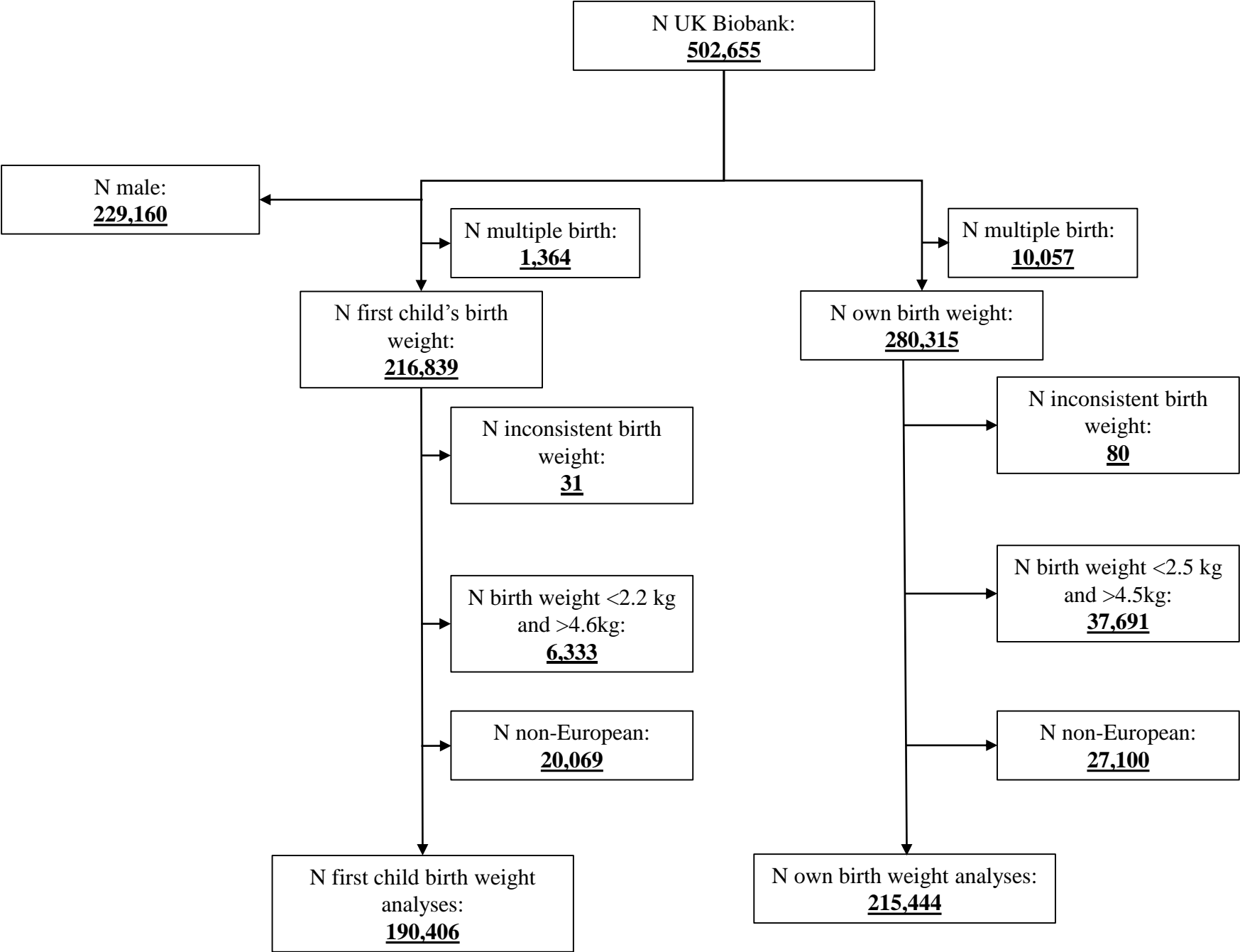

Supplement: S2 Fig — (PDF) [file pmed.1002828.s019.pdf]
