# Supplementary material for: Association of maternal circulating 25(OH)D and calcium with birth weight: A mendelian randomisation analysis
Source: PLoS Med. 2019 Jun 18;16(6):e1002828. doi: 10.1371/journal.pmed.1002828 (PMC6581250; doi:10.1371/journal.pmed.1002828)
Supplement: S3 Fig — RCT, randomised controlled trial. (PDF) [file pmed.1002828.s020.pdf]

S3 Fig: Flow diagram for inclusion of trials in the instrumental variables applied to RCTs

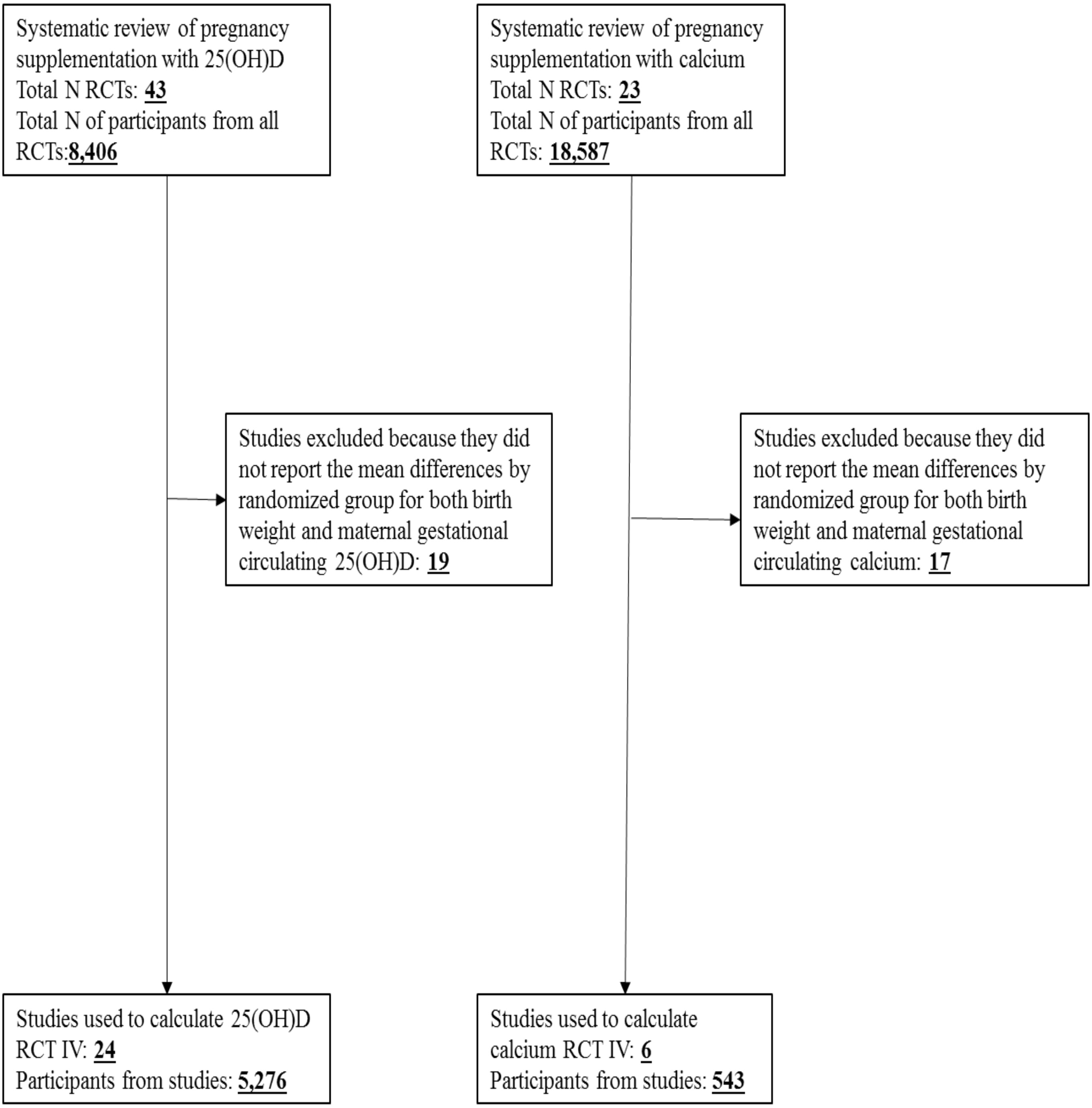

Studies for 25(OH)D were taken from Roth et al 2017[1] and studies for calcium were taken from Buppasiri et al 2015[2].

References

1. Roth DE, Leung M, Mesfin E, Qamar H, Watterworth J, Papp E. Vitamin D supplementation during pregnancy: state of the evidence from a systematic review of randomised trials. BMJ. 2017;359

2. Buppasiri P, Lumbiganon P, Thinkhamrop J, Ngamjarus C, Laopaiboon M, Medley N. Calcium supplementation (other than for preventing or treating hypertension) for improving pregnancy and infant outcomes. Cochrane Database of Systematic Reviews. 2015;(2). doi: 10.1002/14651858.CD007079.pub3.
