# Supplementary material for: Association of maternal circulating 25(OH)D and calcium with birth weight: A mendelian randomisation analysis
Source: PLoS Med. 2019 Jun 18;16(6):e1002828. doi: 10.1371/journal.pmed.1002828 (PMC6581250; doi:10.1371/journal.pmed.1002828)
Supplement: S4 Fig — (PDF) [file pmed.1002828.s021.pdf]

S4 Fig: Leave-One-Out Analysis for effect of maternal gestational circulating 25(OH)D on birth weight Mendelian randomisation Wald ratio estimate in UK Biobank

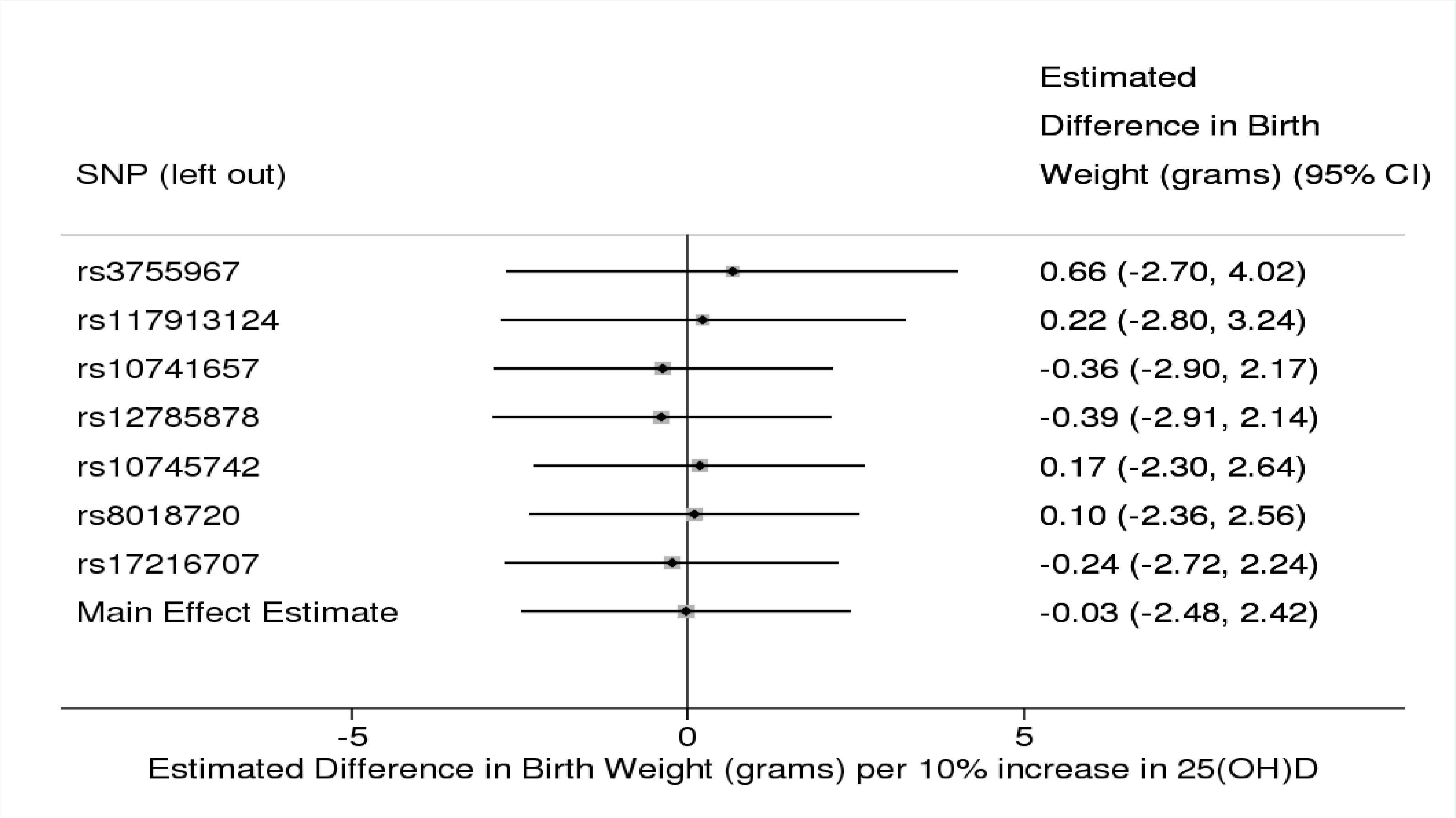

SNPs were taken from Manousaki et al 2017[1] and Jiang et al 2018[2].

References

1. Manousaki D, Dudding T, Haworth S, Hsu Y-H, Liu C-T, Medina-Gómez C, et al. Low-Frequency Synonymous Coding Variation in CYP2R1 Has Large Effects on Vitamin D Levels and Risk of Multiple Sclerosis. The American Journal of Human Genetics. 2017;101(2):227-38. doi: https://doi.org/10.1016/j.ajhg.2017.06.014.

2. Jiang X, O'Reilly PF, Aschard H, Hsu YH, Richards JB, Dupuis J, et al. Genome-wide association study in 79,366 European-ancestry individuals informs the genetic architecture of 25-hydroxyvitamin D levels. Nature communications. 2018;9(1):260. Epub 2018/01/19. doi: 10.1038/s41467-017-02662-2.
