# Supplementary material for: Association of maternal circulating 25(OH)D and calcium with birth weight: A mendelian randomisation analysis
Source: PLoS Med. 2019 Jun 18;16(6):e1002828. doi: 10.1371/journal.pmed.1002828 (PMC6581250; doi:10.1371/journal.pmed.1002828)
Supplement: S5 Fig — RCT, randomised controlled trial. (PDF) [file pmed.1002828.s022.pdf]

S5 Fig: Leave-One-Out Analysis for effect of 25(OH)D on birth weight RCT instrumental variable Wald ratio estimate

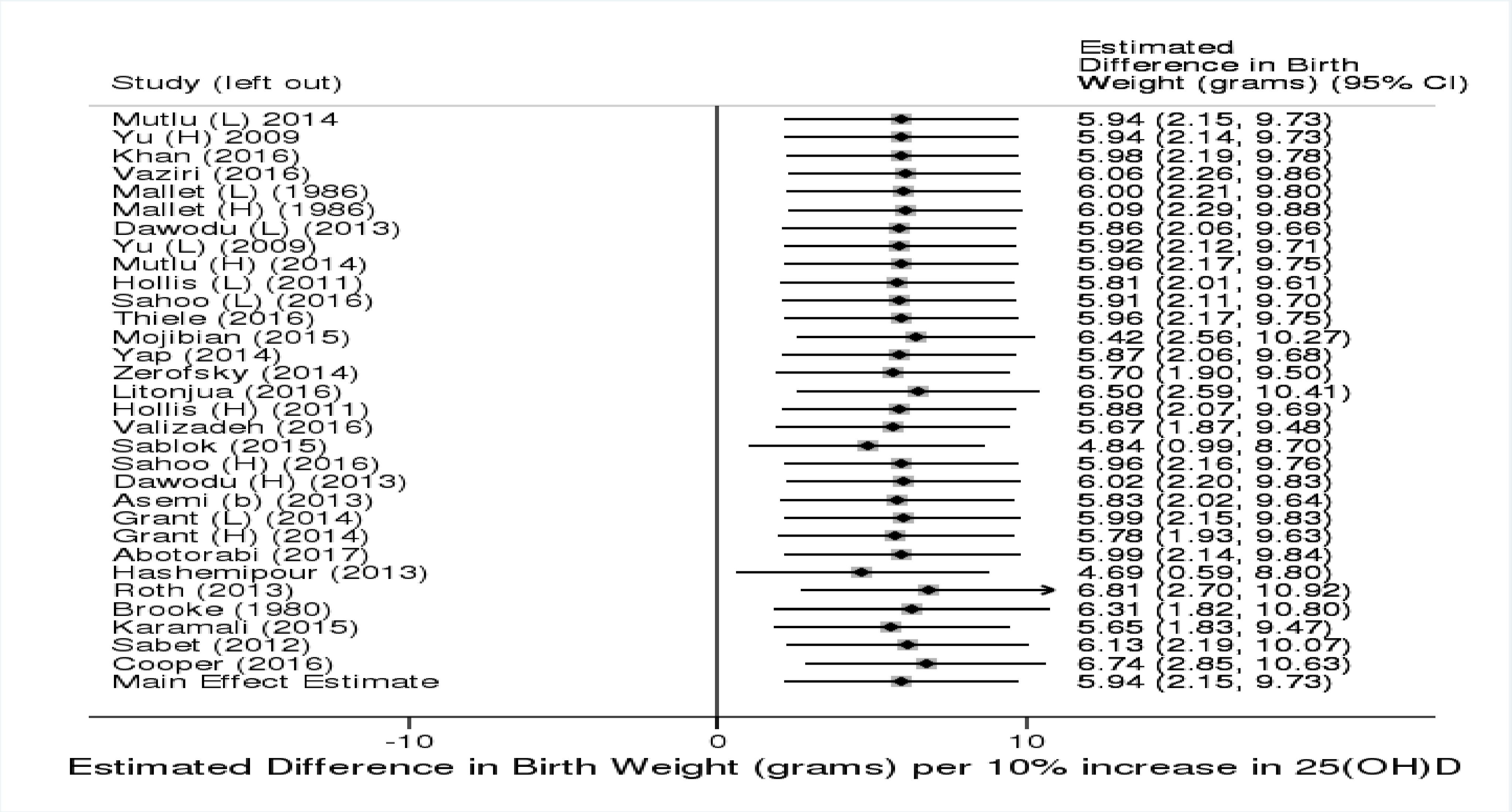

Studies were taken from Roth et al 2017[1].

References

1. Roth DE, Leung M, Mesfin E, Qamar H, Watterworth J, Papp E. Vitamin D supplementation during pregnancy: state of the evidence from a systematic review of randomised trials. BMJ. 2017;359.
