# Supplementary material for: Association of maternal circulating 25(OH)D and calcium with birth weight: A mendelian randomisation analysis
Source: PLoS Med. 2019 Jun 18;16(6):e1002828. doi: 10.1371/journal.pmed.1002828 (PMC6581250; doi:10.1371/journal.pmed.1002828)

S6 Fig: Mendelian randomisation effect estimates for maternal 25(OH)D synthesis and metabolism on birth weight in UK Biobank

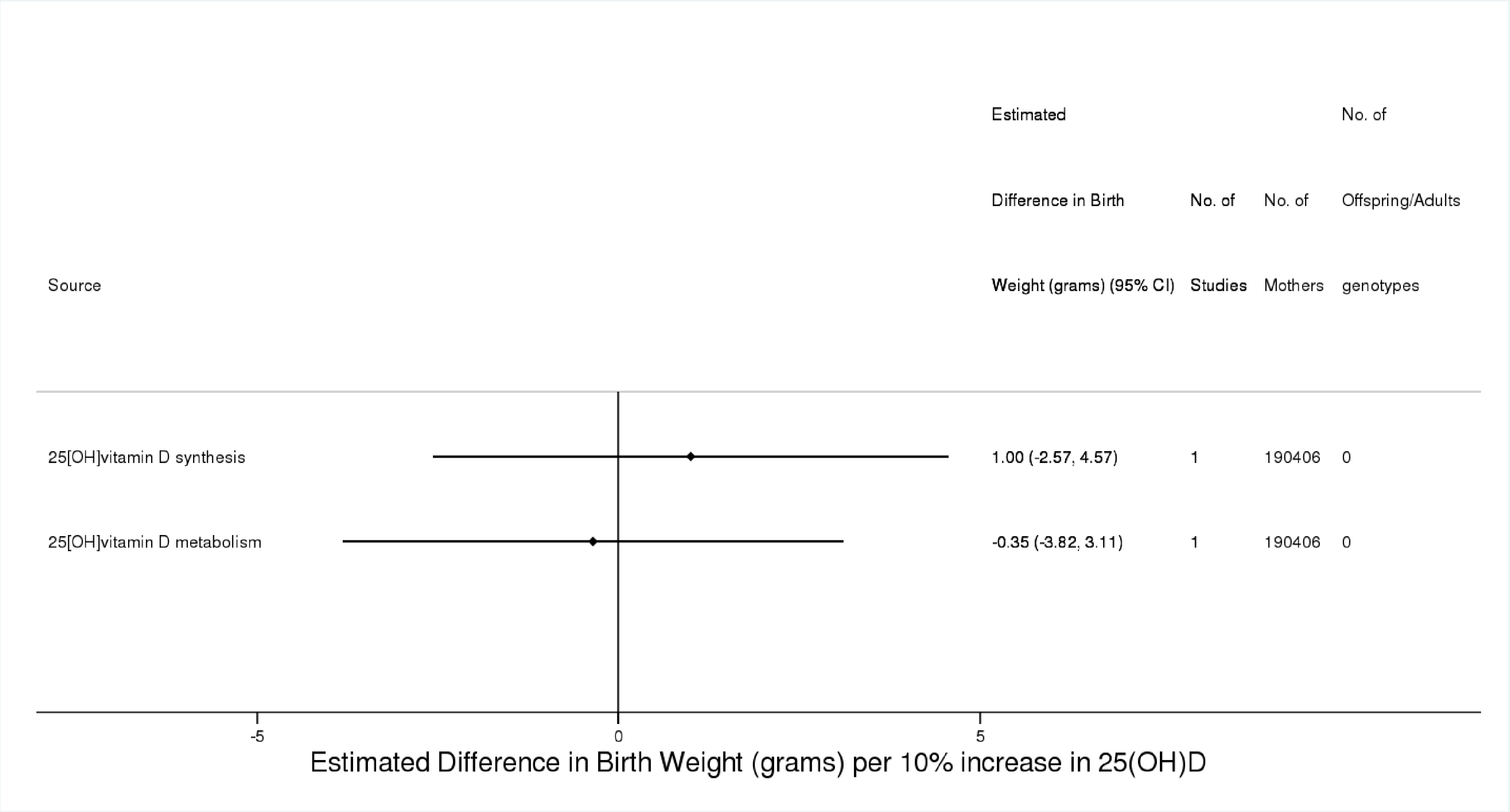

Supplement: S6 Fig — (PDF) [file pmed.1002828.s023.pdf]
