# Supplementary material for: Association of maternal circulating 25(OH)D and calcium with birth weight: A mendelian randomisation analysis
Source: PLoS Med. 2019 Jun 18;16(6):e1002828. doi: 10.1371/journal.pmed.1002828 (PMC6581250; doi:10.1371/journal.pmed.1002828)
Supplement: S7 Fig — (PDF) [file pmed.1002828.s024.pdf]

S7 Fig: Leave-One-Out Analysis for effect of maternal gestational circulating calcium on birth weight Mendelian randomisation Wald ratio estimate in UK Biobank

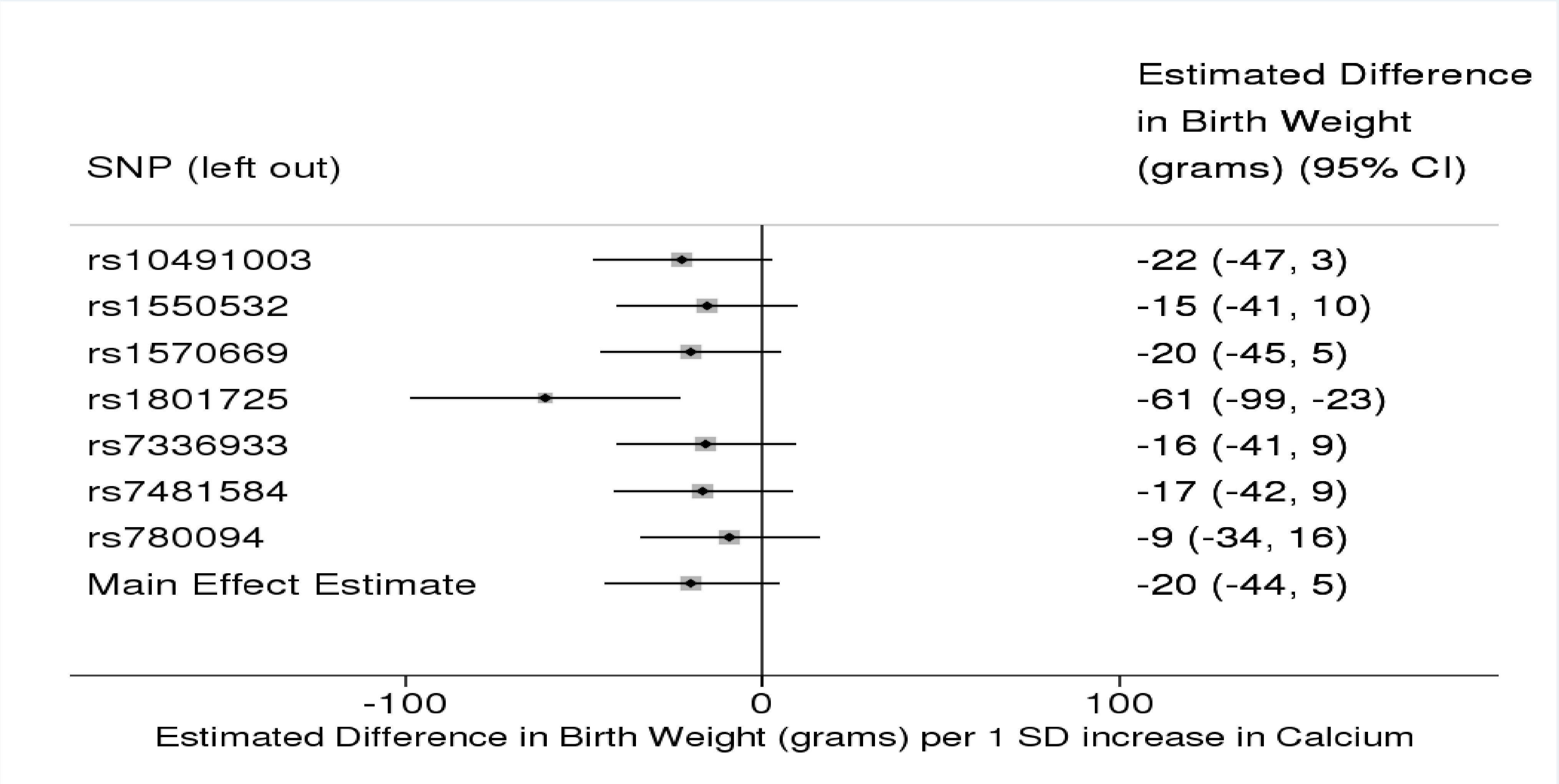

SNPs were taken from O'Seaghdha et al 2013[1]

References

1. O'Seaghdha CM, Wu H, Yang Q, Kapur K, Guessous I, Zuber AM, et al. Meta-Analysis of Genome-Wide Association Studies Identifies Six New Loci for Serum Calcium Concentrations. PLOS Genetics. 2013;9(9):e1003796. doi: 10.1371/journal.pgen.1003796.
