# Supplementary material for: Association of maternal circulating 25(OH)D and calcium with birth weight: A mendelian randomisation analysis
Source: PLoS Med. 2019 Jun 18;16(6):e1002828. doi: 10.1371/journal.pmed.1002828 (PMC6581250; doi:10.1371/journal.pmed.1002828)
Supplement: S8 Fig — RCT, randomised controlled trial. (PDF) [file pmed.1002828.s025.pdf]

S8 Fig: Leave-One-Out Analysis for effect of maternal gestational circulating calcium RCT instrumental variable Wald ratio estimate

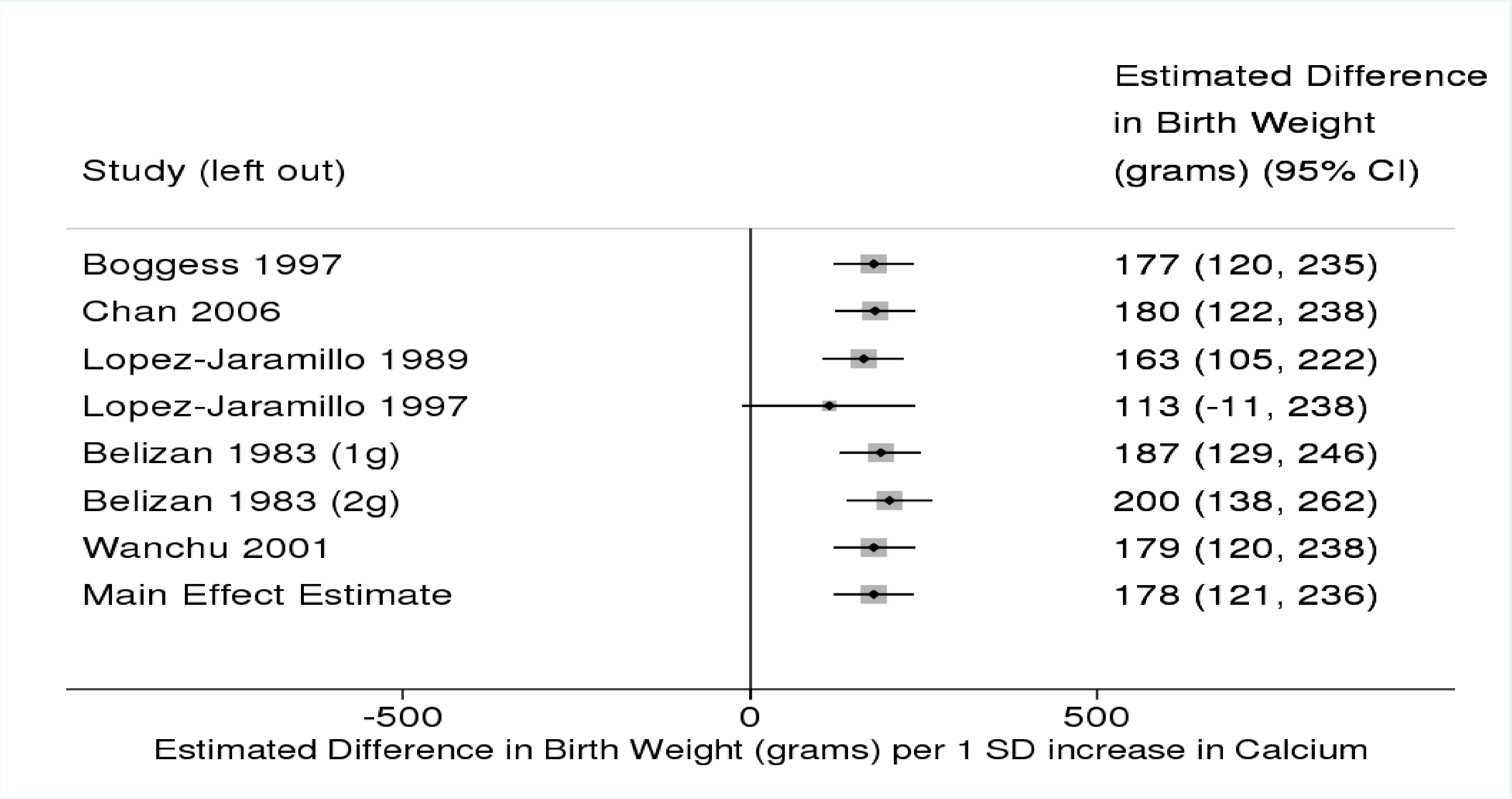

Studies were taken from Buppasiri et al 2015[1].

References

1. Buppasiri P, Lumbiganon P, Thinkhamrop J, Ngamjarus C, Laopaiboon M, Medley N. Calcium supplementation (other than for preventing or treating hypertension) for improving pregnancy and infant outcomes. Cochrane Database of Systematic Reviews. 2015;(2). doi: 10.1002/14651858.CD007079.pub3.
